# Genome-Wide Analysis of U-box E3 Ubiquitin Ligase Family in Response to ABA Treatment in *Salvia miltiorrhiza*

**DOI:** 10.3389/fpls.2022.829447

**Published:** 2022-02-09

**Authors:** Chengan Chen, Can Wang, Junbo Li, Xiankui Gao, Qikai Huang, Yifu Gong, Xiaolong Hao, Itay Maoz, Guoyin Kai, Wei Zhou

**Affiliations:** ^1^Laboratory for Core Technology of TCM Quality Improvement and Transformation, School of Pharmaceutical Sciences, The Third Affiliated Hospital, School of Pharmacy and Academy of Chinese Medical Science, Zhejiang Chinese Medical University, Hangzhou, China; ^2^Department of Postharvest Science, Agricultural Research Organization, Volcani Center, Rishon LeZion, Israel

**Keywords:** *Salvia miltiorrhiza*, genome-wide analysis, ABA treatment, expression pattern, U-box E3 ubiquitin ligases

## Abstract

Plant U-box (PUB) proteins are ubiquitin ligases (E3) involved in multiple biological processes and in response to plant stress. However, the various aspects of the genome and the differences in functions between the U-box E3 (UBE3) ubiquitin ligases remain quite obscure in *Salvia miltiorrhiza*. The 60 *UBE3* genes in the *S. miltiorrhiza* genome were recognized in the present study. The phylogenetic analysis, gene structure, motifs, promoters, and physical and chemical properties of the genes were also examined. Based on the phylogenetic relationship, the 60 *UBE3* genes were categorized under six different groups. The U-box domain was highly conserved across the family of *UBE3* genes. Analysis of the *cis*-acting element revealed that the *UBE3* genes might play an important role in a variety of biological processes, including a reaction to the abscisic acid (ABA) treatment. To investigate this hypothesis, an ABA treatment was developed for the hairy roots of *S. miltiorrhiza*. Thirteen out of the *UBE3* genes significantly increased after the ABA treatment. The co-expression network revealed that nine *UBE3* genes might be associated with phenolic acids or tanshinone biosynthesis. The findings of the present study brought fresh and new understanding to the participation of the *UBE3* gene family in plants, specifically in their biological responses mediated by the ABA. In *S. miltiorrhiza*, this gene family may be crucial during the ABA treatment. Significantly, the results of this study contribute novel information to the understanding of the ubiquitin ligase gene and its role in plant growth.

## Introduction

*Salvia miltiorrhiza* is a well-known Chinese medicinal plant, being widely used for over 1,000 years in medicine and health foods. Many countries have used it in the treatment of cardiovascular and cerebrovascular diseases. Thus far, *S. miltiorrhiza* has served as a model for Chinese herbal medicine due to its comprehensive investigation of its medicinal value. Specifically, the *S. miltiorrhiza* produces two biologically active constituents: phenolic acids (water-soluble) and tanshinones (lipid-soluble) according to Deng et al. (2020); these show a variety of biological activities, such as anti-tumor, anti-inflammatory, and antibacterial outcomes. Consequently, DNA level gene identification (genome analysis, functional gene mining, verification of metabolic pathways, etc.) and RNA transcription level regulation (transcription factor, small RNA mining, and function verification) of *S. miltiorrhiza* were systematic researched (Xu et al., 2016; Wu et al., 2018; Shi et al., 2021). However, only some studies reported the influence exerted by the abiotic/biotic stress or hormone treatment on the post-translational protein levels (Hirayama and Shinozaki, 2010). This indicates that the process of the modification of the ubiquitin protein, as well as the degradation of the functional proteins that manage the metabolic synthesis of the active components, continues to remain unclear in *S. miltiorrhiza*.

Both in eukaryotes and plants, the ubiquitin/26S proteasome system (UPS) pathway is one of the well-investigated mechanisms involved in the post-translational regulation of gene expression (Wang et al., 2018). The UPS is crucial in signal transduction, metabolic regulation, differentiation, cell cycle transition, and stress response regulation, resulting in specific proteins becoming degraded (Vandereyken et al., 2018). The UPS includes three enzymes that exhibit synergistic catalytic action, namely, ubiquitin-activating enzyme (E1), ubiquitin-conjugating enzyme (E2), and ubiquitin ligase (E3) (Trujillo and Shirasu, 2010). The free ubiquitin in the ATP hydrolysis is activated by the E1 enzyme, after which it is transferred to the E2 enzyme. In the presence of E3, ubiquitin is transferred from the E2-ubiquitin intermediate to the substrate protein (Richburg et al., 2014).

The E3 ubiquitin ligase family is the largest and shows the most diversity of the three enzymes catalyzing the ubiquitination cascade. It is a family of proteins that can recognize the target proteins that will be modified, which is the determining factor for specificity of ubiquitination (Serrano et al., 2018). The E3 ubiquitin ligases include several different families according to their action mode and conserved domains, such as the homology to the E6-associated protein C-terminus (HECT), Really Interesting New Gene (RING)/U-box E3 (UBE3) domain, and Cullin-RING ubiquitin ligases (CRLs). An HECT domain, characterized by 350 amino acids, is found on HECT type E3, and its functions to receive ubiquitin from an E2 enzyme and transfer it to a substrate protein (Rotin and Kumar, 2009). So far, *Arabidopsis thaliana* has been analyzed only for HECT type E3s, which contain seven *HECT* genes named UPL1-UPL7 (Downes et al., 2003). The RING/U-box proteins which are dissimilar to the HECT-type E3s, interact non-covalently with the E2 bearing the thioester-linked ubiquitin *via* the conserved RING/U-box domain to enable the ubiquitin to be transferred to the substrate (Callis, 2014). It is possible to use the RING/UBE3 ligase as a single unit for the direct transfer of the ubiquitin to a target protein. Conversely, the multi-component CRLs can function along with the Skp1-Cullin-F-box (SCF) complex (Harper and Tan, 2012; Serrano et al., 2018; Yu et al., 2020). The E3 ubiquitin ligases can either mediate the direct transfer of the ubiquitin to the protein or produce an intermediate complex (E3 and ubiquitin) prior to transfer to the substrate (Stone et al., 2005; Hua and Vierstra, 2011).

UBE3 ubiquitin ligases are a recently discovered subfamily of RING ubiquitinases, containing 70 amino acids residues in the U-box domain (Aravind and Koonin, 2000; Cyr et al., 2002). In comparison with the RING domain, the histidine and cysteine zinc ion chelating residues are absent in the U-box domain. Using the intramolecular interactions, the UBE3-ubiquitin ligases stabilize the U-box scaffolding employing the hydrogen bonds (Kraft et al., 2005; Yee and Goring, 2009). Besides, a domain of the U-box acts as the binding site where the E3-ubiquitin ligase binds with the E2 enzyme. A variety of UBE3-ubiquitin ligases have been identified in several plant genomes. In *Arabidopsis thaliana*, predictions of above 60 *UBE3* genes were made (Cho et al., 2008); other predictions include 77 *UBE3* genes in *Oryza sativa* (Zeng et al., 2008), 56 in Grapevine (*Vitis vinifera L.*) (Yu et al., 2016), 62 in *Solanum lycopersicum* (Sharma and Taganna, 2020), 91 in *Musa acuminate* (Hu et al., 2018), and 125 in Soybean [*Glycine max (L.) Merr.*] (Wang et al., 2016). When the *Arabidopsis PUB*13 was inactivated, it resulted in spontaneous cell death, escalation in salicylic acid (SA) the defense hormone, and early flowering (Trujillo, 2018). The *PUB* gene regulated the accumulation of resistance proteins in Grapevine both in response to biotic and abiotic stressors (Jiao et al., 2017). In Soybean, researchers produced two different *GmPUB8*-overexpressing lines from transgenic *Arabidopsis* plants and found that the heterogeneous over-expression of the *GmPUB8* in *Arabidopsis* resulted in the inhibition of ABA-mediated stomatal closure. The genes connected with drought stress were induced to a lower degree in the *GmPUB8*-overexpression in *Arabidopsis* post the treatment involving exposure to drought (Wang et al., 2016). Hence, from all these findings it appears that the UBE3 proteins may play a fundamental part in the regulation of a variety of biological processes, like hormone-signaling regulations, self-incompatibility, seed germination, and flowering time, as well as in abiotic/biotic stress (Kong et al., 2015; Ma et al., 2015; Trujillo, 2018).

To date, the dissection of the *S. miltiorrhiza* genome in its entirety offers a good platform for molecular biology to analyze its gene family, functional gene mining, and genome evolution. However, the *UBE3* gene family in *S. miltiorrhiza* has not yet been thoroughly examined. As these *UBE3* genes may be crucial in the regulation of the growth and development, it is imperative to fully examine the UBE3-ubiquitin ligase family in *S. miltiorrhiza*. In the present work, the *UBE3* gene number, gene structure, conserved domains, subgroup classification and co-expression analysis were systematically investigated in the entire genome of *S. miltiorrhiza*. Further, the expression profiles of genes in different tissues were studied undergoing the ABA treatment, thus giving a valuable reference for the functional recognition of the *UBE3* genes.

## Materials and Methods

### Sequence Retrieval and Characterization

To look for potential UBE3 ubiquitin ligases, a search was conducted of the database on the *S. miltiorrhiza* genome (Xu et al., 2016)^1^. From the Pfam database, it was possible to find the seed file of the U-box domain (PF04564). Utilizing the HMMER program, the potential UBE3 ubiquitin ligase members in *S. miltiorrhiza* were identified (Finn et al., 2011). All of the UBE3 protein candidates, drawn from the HMM search, were then submitted to the SMART website^2^ in order to ascertain the conserved domain of the U-box. The physical locations of *UBE3* genes on scaffold were obtained from *S. miltiorrhiza* genome database and visualized by TBtools software^3^. Besides, the number of amino acids, molecular weight, theoretical pI, instability index, aliphatic index, and GRAVY (Grand average of hydropathicity) were calculated using the ExPASy-Compute pI/Mw tool (Gasteiger et al., 2005). Cell PLoc 2.0 was used to predict the subcellular localization of the *UBE3* candidate genes^4^ (Emanuelsson et al., 2000).

### Phylogenetic Tree Construction

In the present work, the UBE3 protein sequences were collected for *A. thaliana*^5^, *S. lycopersicum*^6^, and *S. miltiorrhiza*. Multiple alignments of 183 UBE3 protein sequences were performed using MEGA 6.0 software (Tamura et al., 2013). The neighbor-joining (NJ) method was employed to construct the phylogenetic tree, with 1,000 replications as the bootstrap value. The phylogenetic tree of the UBE3 proteins was edited by introducing an Interactive Tree of Life (iTOL) as shown by Letunic and Bork (2021).

### Gene Structure and Conserved Motif Analysis

The *UBE3* genes in *S. miltiorrhiza* were identified and visualized in terms of the structural organization (coding domain sequences and untranslated regions) using the GSDS (gene structure display system) TOOL^7^ (Hu et al., 2015). The conserved motifs of the *UBE3* genes were obtained utilizing the MEME suite^8^, as shown by Bailey et al. (2009). Ten MEME motifs, ranging from 6 to 50 amino acids, were used in this study. All the results of the gene structure and conserved motifs were visualized using the TBtools software (Chen et al., 2020).

### Promoter and Gene Ontology Analysis

The putative promoter sequences refer to 3,000 bp long upstream of the initiation codon. Sixty promoter sequences of *UBE3* genes were extracted using TBtools. PlanCare predicted the *cis*-acting regulatory elements of the promoter sequences (Rombauts et al., 1999). Based on the functional annotations of the *cis*-acting elements, the candidate elements were identified for more investigation. Those *cis*-acting elements possessing similar functional annotations were classified under the same class. Employing the WordArt tool^9^, the word art image of the *cis*-acting elements present in the promoters was generated. From the PANTHER database, the ontology information was drawn and visualized (Mi et al., 2005).

### Hairy Root Treated With ABA Treatment, Illumina Sequencing and *de novo* Transcriptome Assembly

The first step was the cultivation of the sterile *S. miltiorrhiza* plants on the Murashige and Skoog (MS) media keeping the temperature at 25°C, and under a photoperiod of 16 h light/8 h darkness (Zhou et al., 2021). Next, cultures of the *A. tumefaciens* strain C58C1 (pRiA4) were used to infect the *S. miltiorrhiza* sterile stems and/or leaves to produce hairy roots (Shi et al., 2016). The various treatments with ABA were performed on the well-developed hairy roots of *S. miltiorrhiza*. After 0, 0.5, 1, 2, 4, and 8 h of treatment, respectively, these hairy roots were selected to undergo RNA isolation and cDNA synthesis.

Employing the cDNA Synthesis Kit (Clontech, United States) and adopting the protocols prescribed, reverse transcription was done. Once separation was done of the double-stranded cDNAs on agarose gel, they were examined for the RNA-seq. The cDNA library was constructed using the Majorbio Bio-pharm Technology (Shanghai, China) and sequencing was done by Illumina HiSeq TM 2500 with PE100. In fact, all the reads were uploaded in the public database of the National Center for Biotechnology Information (NCBI) under the SRA access number SRP307198. As cited prior, the *de novo* assembly of the Illumina-sequenced short-length reads was accomplished (Zhou et al., 2017). A co-expression network of all *UBE3* genes between important phenolic acids or tanshinone biosynthetic genes was performed by Cytoscape software (Pearson correlation coefficient *r* > 0.8 and *p*-value < 0.05) (Shannon et al., 2003). Our analysis of RNA-seq data yielded FPKM values for quantification of gene expression, and we visualized our results using TBtools.

### Gene Expression Profiles Detected by Quantitative Real-Time PCR

An RNA prep Pure Plant kit (Tiangen Biotech Co., Ltd., Beijing, China) was employed as a simple and inexpensive method of purifying the total RNA resulting from the treatment of different plant tissues (root, stem, leaf, and flower) and hairy roots with ABA, then subjected to RNA isolation and the RNA samples were reverse transcribed to cDNA (Zhou et al., 2021). After synthesizing the cDNA of each sample, a quantitative real-time PCR (qRT-PCR) assay was performed using a SuperReal PreMix Plus (SYBR Green) kit (Tiangen, China), on the ABI StepOnePlus Real-Time PCR System (Applied Biosystems, United States) as explained earlier (Mi et al., 2005). For an internal control in this study, the *SmActin* gene was used. Supplementary Table 1 shows a summary of the primer pairs for qRT-PCR. The 2^–ΔΔ^ ^Ct^ quantification technique of gene expression was utilized (Shi et al., 2016). Three independent experiments were used to determine every data point.

## Results

### Identification and Characterization of *UBE3* Gene Family

The HMMER tool with default parameters enabled the identification of 66 *UBE3* candidate genes in the whole genome of *S. miltiorrhiza*. The SMART tool was used to confirm that U-box domains existed on 66 *UBE3* candidate genes. Among them, 6 *UBE3* genes lacking the U-box domain were removed. Finally, a selection of 60 *UBE3* genes possessing the complete U-box domains were done and were categorized as *SmU-box 1*-*60*, respectively, depending upon the scores of *UBE3* genes presented in the HMM search. The *SmU-box* genes ranged from 555 to 9,332 bp in length, and encoded the polypeptides of 184 to 1,376 amino acids (aa), having a calculated molecular mass of 20 to 152.9 kDa, with 5.19 to 10.04 as the predicted isoelectric point (Supplementary Table 2). The sixty *SmU-box* genes were randomly and unequally distributed in the scaffold of the *S. miltiorrhiza* genome. Among them, scaffold 1254, 15043, and 7427 were the most distributed, with two *UBE3* genes (Supplementary Figure 1). For example, *SmU-box19* and *SmU-box31* on scaffold 1254 were 7,363 bp apart. *SmU-box43* was 37,433 bp away from *SmU-box49*, and *SmU-box43* shared 91.81% identity with *SmU-box49*. *SmU-box2* was 2,119 bp away from *SmU-box36* (Supplementary Figure 1). Interestingly, *SmU-box43* from *SmU-box49* was involved in a tandem duplication event. A majority of the proteins were predicted to be unstable and hydrophilic. From the subcellular localization, it was evident that most of the UBE3 proteins might be found in the nucleus, while the remaining were predicted to be localized either in the plasma membrane or cytosol (Supplementary Table 2).

### Phylogenetic Relationships of the *UBE3* Gene Family Members

**FIGURE 1:**
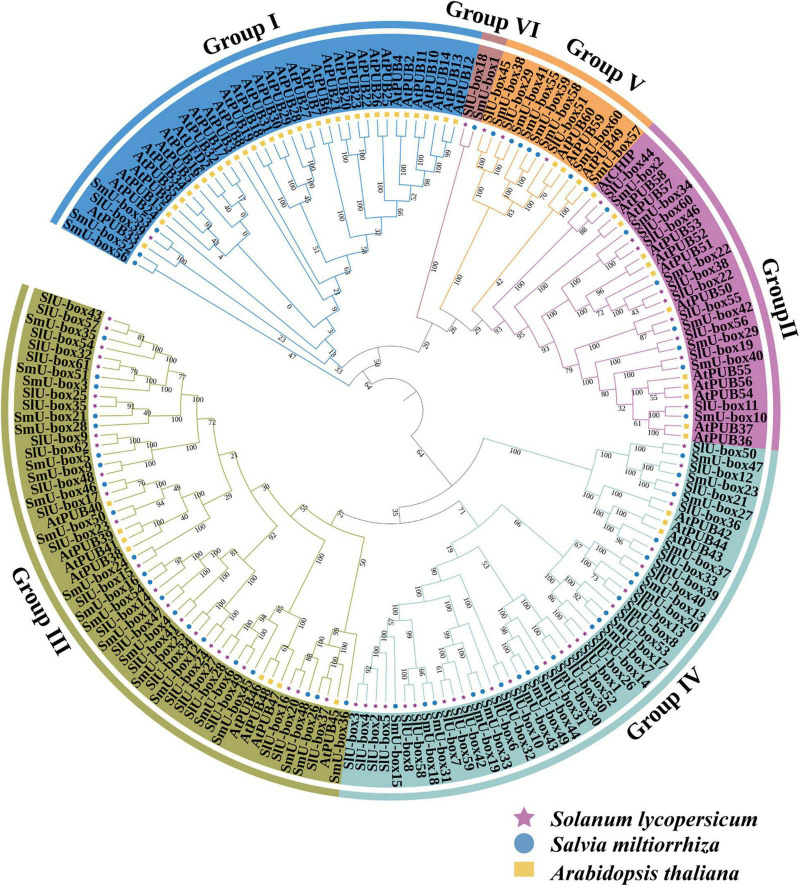
Phylogenetic tree of *S. miltiorrhiza* U-box E3 ubiquitin ligase genes. The phylogenetic tree was constructed by the neighbor-joining method with 1,000 bootstraps. The numbers on the nodes indicate the bootstrap values after 1,000 replicates. *S. miltiorrhiza* U-box gene family was clustered into six subgroups and named Group I-VI. The purple star, yellow square, and blue round represented the U-box proteins in *Solanum lycopersicum*, *Arabidopsis thaliana*, and *S. miltiorrhiza*, respectively.

On the basis of the UBE3 proteins identified from *S. miltiorrhiza* (60 members), *S. lycopersicum* (62 members), and *A. thaliana* (61 members), a phylogenetic tree was drawn to examine the evolutionary history of the *UBE3* genes (Figure 1). From the three species cited earlier, the 183 *UBE3* genes were generally classified under six subgroups (I-VI). Group I included the *SmU-box52*, *53*, and *56*. It was clear that *SmU-box53* and *S. lycopersicum* ubiquitin fusion degradation 39 (*SlU-box39*) had the highest homology, with the amino acid homology of 49.64%. In Group II, each of the seven members contained a serine/threonine kinase domain in the region of the N-terminal. The biggest groups of the *SmU-box* proteins family included Groups III and IV, of which Group III possessed the ARMADILLO (ARM) repetitions at the C-termini. However, Group IV members all possessed around 100 amino acids in the GKL domain (leucine-rich feature) composed of conserved glycine (G), lysine (K), or arginine (R) residues, situated near the C-terminus of the protein (Zeng et al., 2008). Group VI, on the other hand, had the minor *SmU-box* group, which contained only one gene (*SmU-box*1). *SmU-box1* was highly homologous to the *S. lycopersicum* ubiquitin conjugation factor 18 (*SlU-box18*), reaching to the amino acid homology of 42.42%. According to the NCBI annotation, the *SlU-box18* may be a new ubiquitin conjugation factor. In Group V, seven members were presented, containing six WD40 repeat sequences. Generally, the *UBE3* genes of both *S. miltiorrhiza* and *S. lycopersicum* appeared to be clustered as a single subclade in the phylogenetic tree built, indicative of the relatively closer relationship between *S. miltiorrhiza* and *S. lycopersicum* when compared with *Arabidopsis*.

In all three species, the total number of *UBE3* gene families was relatively stable, revealing the conservative traits of *UBE3* genes. In order to evaluate the degree of gene expansion or loss during evolution, the *UBE3* genes in each group were counted. For the *S. miltiorrhiza*, it was found that Groups I–VI contained 3, 7, 21, 21, 7, and 1 *SmUBE3* genes, respectively (Supplementary Table 3). For the *S. lycopersicum*, Groups I, II, III, IV, V, and VI were noted to include 1, 9, 24, 24, 3, and 1 *SlU-box* genes, respectively. However, for *Arabidopsis*, Groups I, II, III, IV, V, and VI revealed 35, 12, 8, 3, 3, and 0 *AtU-box* genes, respectively. In both of the *S. miltiorrhiza* and *S. lycopersicum*, the comparatively consistent number of genes presented in each clade implies the absence of either gene expansion or loss, in these gene families. By contrast, in *S. miltiorrhiza*, Group I revealed rapid gene expansion while Groups III and IV showed two rapid gene losses in comparison to *Arabidopsis*.

### Gene Structure and Motif Analysis of *UBE3* Genes

**FIGURE 2:**
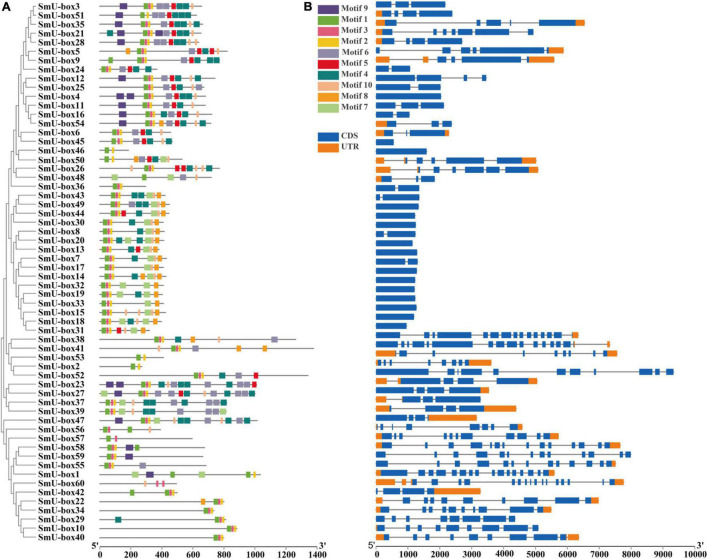
The conserved motifs and gene structure analysis of *U-box* gene family in *S. miltiorrhiza*. **(A)** Distribution of ten motifs in all of the U-box proteins in *S. miltiorrhiza*. A total of 10 motifs were predicated and named motif 1–10. A neighbor-joining (NJ) phylogenetic tree of 60 *S. miltiorrhiza U-box* genes was constructed by Mega 6.0 with 1,000 bootstraps. **(B)** The gene structure of *U-box* genes in *S. miltiorrhiza*. The blue rectangles represented CDS, and the orange rectangles represented UTR.

In order to examine the structure of the exons and introns, we aligned all the full-length cDNA sequences of the *UBE3* genes with the corresponding genome DNA sequences (Figures 2A,B). The exons in the *SmUBE3* genes ranged in number from 1 to 16. In *S. miltiorrhiza* among the 60 *UBE3* genes, the *SmU-box1* and *SmU-box41* revealed the most striking numbers, showing 16 exons, while 15 of the *UBE3* genes (25.0%) possessed only a single exon (Supplementary Table 2). The structural organization was indicative of the wide variations in the *UBE3* genes. This variation in the number of exons may suggest diverse functionality presented within the *UBE3* gene family (*Zhou et al., 2021*).

Furthermore, to identify the conserved motifs, including the UBE3 proteins that were assigned the title motifs 1–10, respectively, the Multiple Em for Motif Elicitation (MEME) was employed (Supplementary Figure 2). The core conservative motifs of the UBE3 proteins were Motifs 1, 2, and 3, while the ARM conventional motifs were the 4, 5, and 6 ones. To date, the characteristics of the motifs 7, 8, 9, and 10 remained unknown. The motifs of the *UBE3* genes that were still unclassified may offer crucial evidence for the biological function that has been recently acquired. The common motifs, however, implied a conserved evolutionary relationship and reveal similar enzyme functions (Nystrom and McKay, 2021). On detailed examination, most of the Group IV members contained only one exon. From this finding, it was evident that the members belonging to the same groups revealed similar gene structures and conserved motifs. It is clear that all of the above solid results validated the conclusions from the phylogenetic tree classifications (Wang et al., 2021).

### Prediction of *Cis*-Acting Elements in Promoter Regions and Gene Ontology Analysis

**FIGURE 3:**
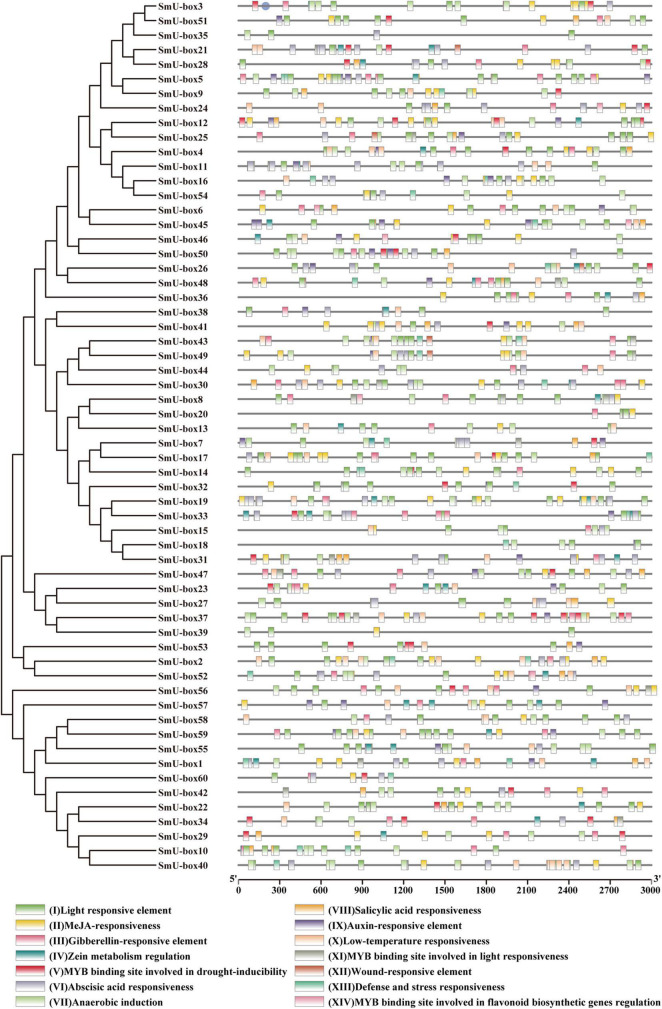
The *cis*-acting elements draft of the putative promoters of 60 *U-box* genes in *S. miltiorrhiza*. The distribution pattern of 14 *cis*-acting elements of the putative promoters of *U-box* genes in *S. miltiorrhiza*. The phylogenetic tree is the same as in Figure 1. Fourteen *cis*-acting elements including (I) Light responsive element; (II) MeJA-responsiveness; (III) Gibberellin-responsive element; (IV) Zein metabolism regulation; (V) MYB binding site involved in drought-inducibility; (VI) Abscisic acid responsiveness; (VII) Anaerobic induction; (VIII) Salicylic acid responsiveness; (IX) Auxin-responsive element; (X) Low-temperature responsiveness; (XI) MYB binding site involved in light responsiveness; (XII)Wound-responsive element; (XIII) Defense and stress responsiveness; (XIV) MYB binding site involved in flavonoid biosynthetic genes regulation.

For greater investigation of the function of the *SmUBE3* genes, the PlantCARE database was introduced to predict the *cis*-acting elements. Finally, 14 *cis*-acting elements linked to stress, hormones, plant growth, and development present in the promoter regions of the 60 *UBE3* genes were selected for greater scrutiny. A diverse distribution pattern was observed in the promoter region of the *SmUBE3* genes, as revealed in Figure 3, the *S. miltiorrhiza UBE3* genes possessed a variety of biological functions. Several common hormone-related *cis*-acting elements were identified in *SmUBE3* genes including abscisic acid (ABA), salicylic acid (SA), gibberellin (GA), auxin, and methyl jasmonate (MeJA) (Supplementary Figure 3). For instance, the presence of an essential *cis*-acting element ABRE observed in response to ABA treatment (Osakabe et al., 2014) was noted in 53 *SmUBE3* genes. This implied that a large number of the *UBE3* genes in *S. miltiorrhiza* may reveal sensitivity to the ABA treatment. In fact, MeJA-responsive elements were recognized in 51 *SmUBE3* genes, and 42 *SmUBE3* genes held *cis*-acting elements related to cold stress, indicating that these genes may possess extraordinary resistance to treatments involving low temperatures. Among the essential plant defense mechanisms, flavonoid biosynthesis is very significant (Falcone Ferreyra et al., 2012). In flavonoid biosynthesis pathways, many R2R3-MYBs serve as activators to regulate the expression of structural genes (Schaart et al., 2013; Xu et al., 2015; Zhai et al., 2016; Zhang et al., 2019). For instance, by promoting the expression of flavonoid structural genes, researchers found that overexpression of *RrMYB5* and *RrMYB10* increased procyanidin accumulation in *Rosa rugosa* and tobacco (Shen et al., 2019). In this work, the MYB binding sites were identified, which participated in flavonoid biosynthesis regulation in 8 promoters of the *SmUBE3* genes (*SmU-box6*, *8*, *24*, *25*, *38*, *45*, *46*, and *52*).

A word cloud image was representative of the frequency of occurrence of the promoter element. In the class of *cis*-acting elements, the following components were recognized: abscisic acid (ABA) responsive elements (ACGTG), anaerobic induction (AAACCA), MeJA-responsive elements (TGACG and CGTCA), low-temperature responsive elements (CCGAAA), light-responsive elements (GGTTAA, CACGTG, and TACGTG), MYB binding site which participates in drought induction (CAACTG), auxin-responsive element (AACGAC), SA responsive element (CCATCTTTTT), gibberellin responsive element (TATCCCA), and stress-responsive elements (ATTCTCTAAC). These elements occurred in abundance in the promoters of the family of the UBE3 ubiquitin ligases. These were related to the abscisic acid response, anaerobic and MeJA induction; especially, the ABA induction had the highest frequency (Figure 3 and Supplementary Figure 4). The large quantities of these regulatory elements in the promoter regions of the *SmUBE3* genes suggested that the *UBE3* gene family had strong associations with the development of the plant and its response to hormone induction.

On analyzing the Gene Ontology (GO) of the 60 *UBE3* genes identified, the Blast2GO software was used to predict the biological process, cellular component, and molecular function (Supplementary Figure 5). A large part of the genes (71.42%) were assigned the GO category of “molecular function,” while the classes of “biological process” and “cellular component” were observed to possess the same lower ratio (14.29%). The *UBE3* genes within the “molecular function” category, were mapped largely to the GO term “ubiquitin-protein ligase activity” (GO:0061630), showing a ratio of 71.42%. The prediction was that all the UBE3 proteins would be present in the nucleus (GO: 0005634) and cytoplasm (GO: 0005737). Further, within the “biological process,” all the proteins were distinguished into protein ubiquitination (GO:0016567), ubiquitin-dependent protein catabolic process (GO:000651), peptidyl-amino acid modification (GO: 0018193), cellular response to misfolded protein (GO:0071218), protein quality control for misfolded or incompletely synthesized proteins (GO:0006515), positive regulation of proteolysis (GO:0045862), proteasome-mediated ubiquitin-dependent protein catabolic process (GO:0043161), protein polyubiquitination (GO:0000209), and ubiquitin-dependent ERAD pathway (GO:0030433). From the gene ontology and promoter analysis, strong evidence was observed to support the relationship of the *UBE3* gene family in *S. miltiorrhiza* with a variety of metabolic pathways.

### Expression Analysis of UBE3 Ubiquitin Ligases Under ABA Treatment

**FIGURE 4:**
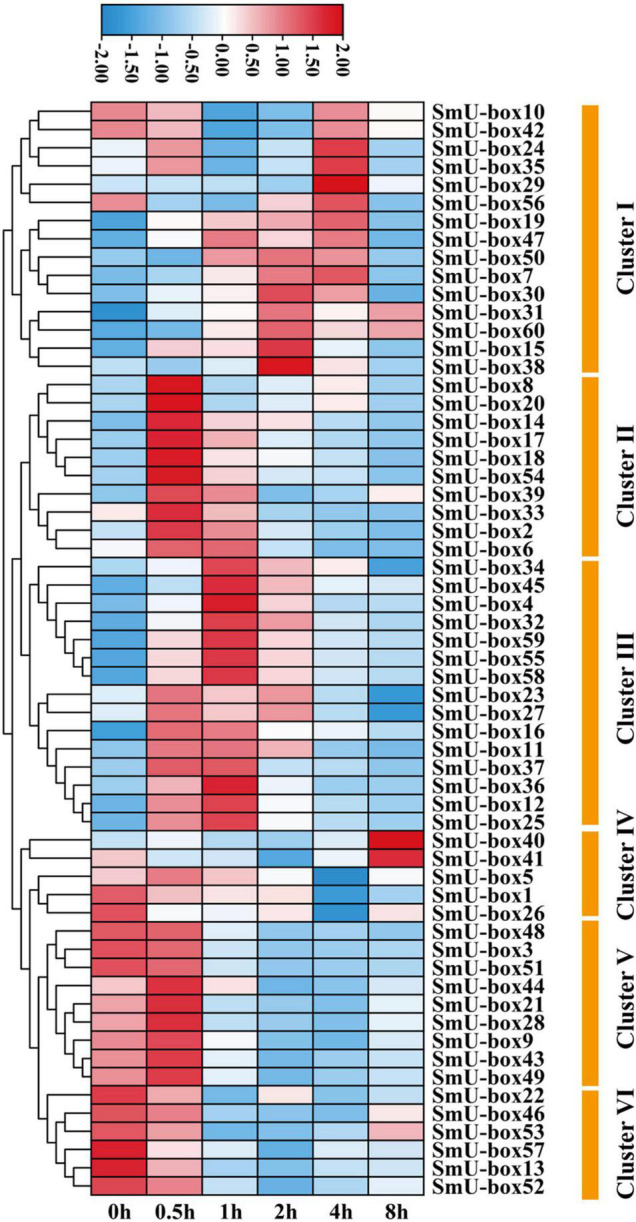
The heat map of *U-box* genes in *S. miltiorrhiza* under ABA treatments based on RNA-seq. Genes with similar expression patterns were clustered into the same group according to the hierarchical clustering method. The top color scale indicated the Fragments Per Kilobases per Million reads (FPKM) values of each gene.

**FIGURE 5:**
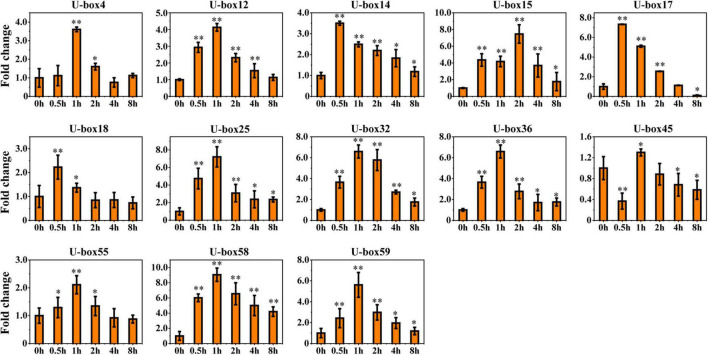
qRT-PCR validation of 13 upregulated genes in response to ABA treatment in RNA-seq dataset. The relative gene expression level changes were normalized to the control without ABA induction at 0 h. Student’s *t*-test was used with two significance levels (**P < 0.01; *P < 0.05), and the data presented the average of three replicates.

The ABA was justified as an abiotic inducer to encourage the biosynthesis of the tanshinones and phenolic acids (medicinal metabolites) in *S. miltiorrhiza* (Deng et al., 2020). The highest quantity of the *cis*-acting elements in the promoter regions of the *UBE3* genes was estimated to be ABA-responsive. In fact, six RNA-seq samples undergoing ABA treatment were collected, and after analysis by RNA sequencing, the expression pattern of the *UBE3* genes was studied in response to this treatment. The value of the Fragments Per Kilobases per Million reads (FPKM) for each of the *UBE3* genes was done to group the expression patterns using the k-means quantization method, it was possible to assemble all of them into six clusters, and those with > 4-fold change were considered as the upregulated *UBE3* genes (Figure 4 and Supplementary Table 4). While Cluster II included three *UBE3* genes, Cluster III contained ten *UBE3* genes which were upregulated by the ABA treatment, with a short inducement time span of 0.5 and 1 h, respectively. Clusters of V and VI showed little or no variation in expression. Ultimately, the qRT-PCR method was used to validate the differential gene expression acquired by the RNA-seq. Under the ABA treatment, 13 *UBE3* genes revealed the highest expression level in comparison to the control; also, *SmU-box4*, *12*, *14*, *15*, *17*, *18*, *25*, *32*, *36*, *45*, *55*, *58*, and *59* were picked up for qRT-PCR testing. After applying the qRT-PCR technique, the expression patterns of the 13 genes acquired showed consistency with the trend of the patterns of expression drawn from RNA-seq data (Figures 4, 5). The correlation between the two data sets was observed to be significant, with the correlation coefficient in the range of 0.9091–0.9916 (Supplementary Figure 6). This established the reliability of the data obtained by RNA-seq for further investigating the *UBE3* candidate genes in response to the ABA treatments.

### Differential Expression of UBE3 Ubiquitin Ligases in Various Tissues

**FIGURE 6:**
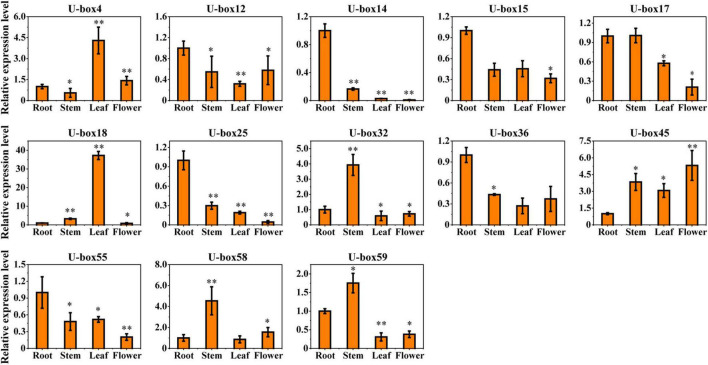
The expression profiles of 13 selected *U-box* genes in different vegetative and reproductive tissues. Asterisks indicate significant differences in the root vs. other tissues by *t*-test with two significant levels (**P < 0.01; *P < 0.05), and the data presented the average of three replicates.

As a means of studying the part they play in plant growth, development, and secondary metabolism biosynthesis of the *S. miltiorrhiza*, 13 profiles of the ABA-responsive *UBE3* genes expressions in the vegetative and reproductive tissues (leaf, stem, root, and flower) were investigated (Figure 6). It was observed that the *SmU-box14*, *18*, and *25* were hard to detect in the reproductive tissues (flower). The *SmU-box4* and 18 uniformly revealed the highest expression in the leaf. Likewise, the *SmU-box32*, *58*, and 59 showed the highest expression in the stem. The *SmU-box12*, *14*, *15*, *17*, *25*, *36*, and *55* revealed the highest gene expression in the root, which in traditional Chinese medicine was utilized as a medicinal material (Zhou et al., 2005, 2021).

### Co-expression Network of *UBE3* Genes With Phenolic Acids and Tanshinone Biosynthetic Genes

Previous studies showed that ABA could promote the phenolic acids and tanshinone accumulation in hairy roots by activating the expression of phenylalanine ammonia-lyase (PAL), tyrosine amino transferase (TAT), and geranylgeranyl diphosphate synthase (GGPPS), respectively (Shi et al., 2021). The co-expression network of 60 *UBE3* genes with phenolic acids and tanshinone biosynthetic genes was constructed, and these results indicated that 9 out of 60 *UBE3* genes showed a correlated relationship with phenolic acids and tanshinone biosynthetic genes (Supplementary Figures 7, 8). Co-expression analysis revealed that six *UBE3* genes (*SmU-box7*, *15*, *19*, *30*, *32*, *50*) showed a positive correlation with *GGPPS* gene in tanshinone biosynthetic pathway with Pearson correlation coefficient (r) > 0.8 and *P*-value < 0.05 as a cutoff (Supplementary Table 5). *SmU-box31* and *SmU-box60* genes were positively correlated with *PAL* gene in phenolic acids biosynthetic pathway (*r* > 0.8). Additionally, five *UBE3* genes (*SmU-box7*, *15*, *30*, *38*, *50*) co-expressed with the *TAT* gene in phenolic acid biosynthetic pathway (*r* > 0.8). Overall, these results suggested that 9 out of 60 *UBE3* genes might participate in phenolic acids and tanshinone biosynthesis.

## Discussion

The U-box E3 (UBE3) ubiquitin ligases show the wide distribution in plants and are well-known as participants in several biological functions (Santner and Estelle, 2010; Tian and Xie, 2013; Duplan and Rivas, 2014). As the *UBE3* genes play a crucial part in plant development, their presence has been recognized in many plant species, such as *A. thaliana* (Cho et al., 2008), rice (Zeng et al., 2008), tomato (Sharma and Taganna, 2020), and banana (Hu et al., 2018). Historically, *S. miltiorrhiza* (family Lamiaceae), one of the most valuable herbal plants extensively used in traditional Chinese medicine, is popular in the treatment of cardiovascular and cerebrovascular diseases (Zhou et al., 2005, 2016; Shi et al., 2016). To further characterize the function of the *UBE3* genes in *S. miltiorrhiza*, identifying these genes in the entire genome would be critical to *S. miltiorrhiza*. However, this field has been poorly studied to date. In this work, the HMMER analysis was done using the Pfam databases and 60 *UBE3* genes were recognized in the *S. miltiorrhiza* genome; it is interesting that the number of the *UBE3* genes in *S. miltiorrhiza* beard similarity to that of *A. thaliana* (61), tomato (62), and pear (62).

From phylogenetic analysis, it was evident that 183 UBE3 protein members in these three species (60 in *S. miltiorrhiza*, 62 in *S. lycopersicum*, and 61 in *A. thaliana*) were classified under six groups I-VI, (Figure 1). The phylogenetic analysis of *UBE3* genes revealed that all the six groups showed good similarity among themselves because the core U-box domain presented in all the genes. The grouping in the phylogenetic tree beard similarity to some other species, like soybean (Wang et al., 2016) and pear (Wang et al., 2021). Thus, the classification based on the phylogenetic relationship implied that when compared with the *PUBs* in *A. thaliana*, the *UBE3* family in *S. miltiorrhiza* demonstrated greater genetic closeness with the *S. lycopersicum*. This result clearly corresponded to the fact that *S. miltiorrhiza* and tomato got a closer relationship than *A. thaliana*. Besides the U-box domain, sixty *SmU-box* proteins were observed to bind to different domains such as the armadillo (ARM) repeats, protein kinase domain, WD40 repeats, GKL-box and the Ufd2P domain. The UBE3 proteins were derived largely from the U-box proteins possessing the ARM repeats (Wang et al., 2020). It is clear that the ARM repeats mostly mediated the interaction between the U-box proteins and substrate, implying that the interaction enabled the substrate to be available for the ubiquitination leading to degradation (Shu and Yang, 2017). The U-box with ARM domain in *S. miltiorrhiza* presented in twenty-one *UBE3* genes. Eight genes possessed the protein kinase domain, while seven genes included the WD40 repeats. In this work, the Ufd2P domain was identified as a single member of the UBE3 ubiquitin ligases family, namely the *SmU-box1*. Due to the differential distribution of these domains in the UBE3 ubiquitin ligases family, diverse biological functions may be inferred (Duplan and Rivas, 2014).

Further, from the structure and organization of the *UBE3* genes, the diversity existent in a gene family within the species is evident, which is related to the evolution and functional differentiation this gene family has experienced (Xu et al., 2012). In fact, from this study, it was evident that several of the *UBE3* genes in *S. miltiorrhiza* possessed either no introns at all or else contained upward of three introns (Figure 2). The general understanding was that a substantial number of introns in the *UBE3* genes behaved like a mutational buffer to ensure that the coding sequence was protected from mutations that are functionally deficient. However, the structural integrity of the gene families was evident from the *UBE3* genes which lacked the introns. All the *UBE3* genes identified were observed to be distributed in 10 classes of motifs. Motifs 1–3 were conserved and revealed good homology with the U-box domain. On the other hand, Motifs 4–6, along with the armadillo-like fold structure, were found to be clustered solely in group III. Hence, in the *S. miltiorrhiza UBE3* genes, the gene structure and motif analysis will be very helpful in predicting both gene evolution and functions for experimental authentication (Bailey et al., 2009).

On analysis of the *cis*-acting elements presented in the promoters, it is understood that the *UBE3* gene families participated in stress-related mechanisms, hormone regulation, growth, and development (Figure 3). In earlier studies, the *UBE3* genes were reported to have the ability to respond to ABA signals. Also, the AtPUB44 ubiquitinated abscission aldehyde oxidase 3 (AAO3) was recognized as a mediator in the ABA biosynthesis through 26 proteasomes (Raab et al., 2009). Besides, in *Arabidopsis*, the regulation of the ABI3 transcription factor was accomplished by the AtPUB9, resulting in heightened sensitivity to the ABA at the time of seedling germination (Samuel et al., 2008). In the case of *Pohlia nutans* (PnSAG1), where the U-box domain included the ARM repeat, the expression was seen to occur rapidly in response to the exogenous ABA (Wang et al., 2019). The AtPUB18, AtPUB19, and AtPUB44 were identified as capable of causing direct interruption of the ABA biosynthesis (Sharma and Taganna, 2020). This study showed that in the putative promoter regions of fifty-three genes, the ABA-responsive elements were presented (Supplementary Figure 3). Specifically, in all the candidate genes, 13 ABA-responsive elements within the promoter region of the *SmU-box17* were observed to be at the maximum. From these results, it can be implied that the *UBE3* genes in *S. miltiorrhiza* may have a vital part to play in the ABA signal transduction process. In *Arabidopsis* and *Nicotiana*, it was the abiotic and biotic stresses which controlled the expression of the *PUB* gene (Raab et al., 2009). Earlier studies indicated that the expression of the resistant genes were regulated by the MYB transcription factor. For example, the PbrMYB21 was able to bind specifically to the MYB recognition sites in the *PbrADC* promoters and positively aid in drought tolerance (Li et al., 2017). In this work, many MYB transcription factor binding sites were recognized, limited to the promoter regions of the *UBE3* genes, and which were linked to drought induction resistance. Therefore, we reasonably concluded, that *UBE3* genes might most likely be regulated by the MYB genes concerned, which mediate the signaling of drought stress. In *S. miltiorrhiza*, the promoter region of *UBE3* genes gave supporting evidence for the presence of the defense and stress-responsive elements, low-temperature responsive elements, and wound-responsive elements. Therefore, it is understood that the *UBE3* genes could play a vital role in a variety of biological processes during the growth and development of *S. miltiorrhiza*.

It is assumed that the *UBE3* genes could take part in several events of abiotic stress and hormone induction (Liu et al., 2011; Seo et al., 2012; Song et al., 2017). Due to the frequent presence of the ABA-responsive elements in the promoter regions of the *SmUBE3* genes (Supplementary Figure 4), this study was conducted to examine the expression pattern of the *UBE3* genes which exhibited a response to the ABA treatment. From the expression profiles, it was possible to gain a clear picture of the potential participation of the *UBE3* genes in response to the ABA. In 13 of the fifty-three *UBE3* genes, it was possible to identify the ABA-responsive elements and validate their expression. Our data concurred with the earlier studies which reported the response of the *UBE3* genes to ABA induction (Seo et al., 2012). Further, the analysis of the gene expression corresponded to the prediction by the *cis*-elements of the *UBE3* gene family. Manifold elements were related to hormone regulation, not restricted to the ABA-responsive elements only, but were inclusive of the SA, GA, MeJA, and auxins (Figure 3). *SnRK2.6* identified as a phosphorylated protein, is considered to be a key gene involved in ABA signaling (Fujii et al., 2009; Melcher et al., 2009; Park et al., 2009; Umezawa et al., 2010). In *S. miltiorrhiza*, a recent study has revealed that *SmSnRK2.6* and *SmAREB1* genes were strongly induced by exogenous ABA. Meanwhile, SmSnRK2.6 protein interacted with SmAREB1 transcription factor. Overexpressing of *SmSnRK2.6* and *SmAREB1* coregulated the rosmarinic acid (RA) and salvianolic acid B (Sal B) accumulation in the transgenic *S. miltiorrhiza* hairy roots. This provided a novel insight on how exogenous ABA promoted the secondary metabolism at the post-translational level in *S. miltiorrhiza* (Jia et al., 2017).

Reports from earlier studies described the expression of the *UBE3* genes in a variety of tissues (Sharma and Taganna, 2020; Wang et al., 2021). In the present study, an investigation was done on the differential levels of expression of 13 *UBE3* genes in several vegetative and reproductive tissues employing the qRT-PCR technique (Figures 5, 6). As the expression of the *UBE3* genes in the *S. miltiorrhiza* is higher in the roots than in the stem, flowers, and leaf, this implied that these genes could have a part to play in the root system formation and thus influence the synthesis of biologically active constituents. On the other hand, the poorest expression was noted in the flowers, indicative that some of these *UBE3* genes could be involved in the growth and development of *S. miltiorrhiza* during exposure to the ABA stress. Additionally, the analysis of co-expression network between *UBE3* genes and phenolic acids or tanshinone biosynthetic genes indicated that 9 *UBE3* genes might be associated with biosynthesis. Our findings show that the *UBE3* genes have a significant spatial association with development and plant growth in *S. miltiorrhiza*.

By way of conclusion, in this study, analyses had been done of the *UBE3* gene family in *S. miltiorrhiza* taking into account the whole genome and transcriptome level. This research presented firstly a systematic and comprehensive analysis of *UBE3* genes in *S. miltiorrhiza*. However, a proper understanding of the functional mechanism of the *UBE3* genes continues to be very meager. Still, this work offers valuable information that will encourage a more detailed understanding of the relationship between the *UBE3* genes and the ABA responses in the future. This paper may assist in a better comprehension of the biological functions of the *UBE3* genes in other plant species, under conditions of abiotic and biotic stress.

## Data Availability Statement

All reads of RNA-seq are publicly available in the National Center for Biotechnology Information (NCBI) database under the SRA accession number SRP307198.

## Author Contributions

WZ and GK formulated the study concept. CC collected the public data available on *S. miltiorrhiza* and the other species in this research. CC, CW, JL, XG, and QH accomplished the analyses of data and bioinformatics and preparation of the manuscript. WZ, GK, YG, and IM contributed to analysis planning and manuscript revision. All authors read and approved the final version of the manuscript.

## Conflict of Interest

The authors declare that the research was conducted in the absence of any commercial or financial relationships that could be construed as a potential conflict of interest.

## Publisher’s Note

All claims expressed in this article are solely those of the authors and do not necessarily represent those of their affiliated organizations, or those of the publisher, the editors and the reviewers. Any product that may be evaluated in this article, or claim that may be made by its manufacturer, is not guaranteed or endorsed by the publisher.

## Footnotes

1
ftp://danshen.ndctcm.org:10402/Salvia_genome.fas

2
http://smart.embl-heidelberg.de/

3
https://github.com/CJ-Chen/TBtools

4
http://www.csbio.sjtu.edu.cn/bioinf/plant-multi/

5
https://www.arabidopsis.org/

6
ftp://ftp.solgenomics.net/tomato_genome/

7
http://gsds.gao-lab.org/

8
http://meme-suite.org/tools/meme

9
https://wordart.com

## Supplementary Material

Supplementary Figure 1 The location of *UBE3* genes in *S. miltiorrhiza* genome.

Supplementary Figure 2 Ten motifs identified using MEME suit for *U-box* gene family.

Supplementary Figure 3 The number of 14 *cis*-acting elements in each putative promoter of *UBE3* genes. The color scale at the bottom indicated the number of *cis*-acting elements.

Supplementary Figure 4 The word cloud image of *cis*-acting elements in the promoter of 60 *UBE3* genes.

Supplementary Figure 5 Gene ontology analysis of 60 *UBE3* genes in *S. miltiorrhiza*. **(A)** Gene ontology analysis of the identified *UBE3* genes in *S. miltiorrhiza*. **(B)** The molecular function of *UBE3* genes in *S. miltiorrhiza*. **(C)** The biological process of *UBE3* genes in *S. miltiorrhiza*. **(D)** The cellular component of *UBE3* genes in *S. miltiorrhiza*.

Supplementary Figure 6 Correlation of gene expression results obtained from qRT-PCR and RNA-seq.

Supplementary Figure 7 The co-expression network of *UBE3* genes and phenolic acids biosynthetic genes.

Supplementary Figure 8 The co-expression network of *UBE3* genes and tanshinone biosynthetic genes.

## Abbreviations

PUB: Plant U-box
U-box E3: UBE3
ABA: abscisic acid
SA: salicylic acid
GA: gibberellin
MeJA: methyl jasmonate.

## References

[B1] Aravind L. Koonin E. V. (2000). The U box is a modified RING finger-a common domain in ubiquitination. *Curr. Biol*. 10 R132–R134. 10.1016/s0960-9822(00)00398-5 10704423

[B2] Bailey T. L. Boden M. Buske F. A. Frith M. Grant C. E. Clementi L. (2009). MEME SUITE: tools for motif discovery and searching. *Nucleic Acids Res.* 37 W202–W208. 10.1093/nar/gkp335 19458158 PMC2703892

[B3] Callis J. (2014). The ubiquitination machinery of the ubiquitin system. *Arabidopsis Book/Am. Soc. Plant Biol*. 12:e0174. 10.1199/tab.0174 25320573 PMC4196676

[B4] Chen C. Chen H. Zhang Y. Thomas H. R. Frank M. H. He Y. (2020). TBtools: an integrative toolkit developed for interactive analyses of big biological data. *Mol. Plant* 13 1194–1202. 10.1016/j.molp.2020.06.009 32585190

[B5] Cho S. K. Ryu M. Y. Song C. Kwak J. M. Kim W. T. (2008). *Arabidopsis* PUB22 and PUB23 are homologous U-Box E3 ubiquitin ligases that play combinatory roles in response to drought stress. *Plant Cell* 20 1899–1914. 10.1105/tpc.108.060699 18664614 PMC2518226

[B6] Cyr D. M. Höhfeld J. Patterson C. (2002). Protein quality control: u-box-containing E3 ubiquitin ligases join the fold. *Trends Biochem. Sci.* 27 368–375. 10.1016/s0968-0004(02)02125-4 12114026

[B7] Deng C. Shi M. Fu R. Zhang Y. Wang Q. Zhou Y. (2020). ABA-responsive transcription factor bZIP1 is involved in modulating biosynthesis of phenolic acids and tanshinones in *Salvia miltiorrhiza*. *J. Exp. Bot*. 71 5948–5962. 10.1093/jxb/eraa295 32589719

[B8] Downes B. P. Stupar R. M. Gingerich D. J. Vierstra R. D. (2003). The HECT ubiquitin-protein ligase (UPL) family in *Arabidopsis*: UPL3 has a specific role in trichome development. *Plant J.* 35 729–742. 10.1046/j.1365-313x.2003.01844.x 12969426

[B9] Duplan V. Rivas S. (2014). E3 ubiquitin-ligases and their target proteins during the regulation of plant innate immunity. *Front. Plant Sci.* 5:42. 10.3389/fpls.2014.00042 24592270 PMC3923142

[B10] Emanuelsson O. Nielsen H. Brunak S. Von Heijne G. (2000). Predicting subcellular localization of proteins based on their N-terminal amino acid sequence. *J. Mol. Biol.* 300 1005–1016. 10.1006/jmbi.2000.3903 10891285

[B11] Falcone Ferreyra M. L. Rius S. Casati P. (2012). Flavonoids: biosynthesis, biological functions, and biotechnological applications. *Front. Plant Sci.* 3:222. 10.3389/fpls.2012.00222 23060891 PMC3460232

[B12] Finn R. D. Clements J. Eddy S. R. (2011). HMMER web server: interactive sequence similarity searching. *Nucleic Acids Res.* 39 W29–W37. 10.1093/nar/gkr367 21593126 PMC3125773

[B13] Fujii H. Chinnusamy V. Rodrigues A. Rubio S. Antoni R. Park S. Y. (2009). In vitro reconstitution of an abscisic acid signalling pathway. *Nature* 462 660–664. 10.1038/nature08599 19924127 PMC2803041

[B14] Gasteiger E. Hoogland C. Gattiker A. Wilkins M. R. Appel R. D. Bairoch A. (2005). “Protein identification and analysis tools on the ExPASy server,” in *The Proteomics Protocols Handbook*, (Berlin: Springer), 571–607. 10.1385/1-59259-890-0:571

[B15] Harper J. W. Tan M. K. M. (2012). Understanding cullin-RING E3 biology through proteomics-based substrate identification. *Mol. Cell. Proteomics* 11 1541–1550. 10.1074/mcp.R112.021154 22962057 PMC3518111

[B16] Hirayama T. Shinozaki K. (2010). Research on plant abiotic stress responses in the post-genome era: past, present and future. *Plant J.* 61 1041–1052. 10.1111/j.1365-313X.2010.04124.x 20409277

[B17] Hu B. Jin J. Guo A. Y. Zhang H. Luo J. Gao G. (2015). GSDS 2.0: an upgraded gene feature visualization server. *Bioinformatics* 31 1296–1297. 10.1093/bioinformatics/btu817 25504850 PMC4393523

[B18] Hu H. Dong C. Sun D. Hu Y. Xie J. (2018). Genome-wide identification and analysis of U-box E3 ubiquitin-protein ligase gene family in banana. *Int. J. Mol. Sci.* 19:3874. 10.3390/ijms19123874 30518127 PMC6321073

[B19] Hua Z. Vierstra R. D. (2011). The cullin-RING ubiquitin-protein ligases. *Annu. Rev. Plant Biol*. 62 299–334. 10.1146/annurev-arplant-042809-112256 21370976

[B20] Jia Y. Bai Z. Pei T. Ding K. Liang Z. Gong Y. (2017). The protein kinase *SmSnRK2.6* positively regulates phenolic acid biosynthesis in *Salvia miltiorrhiza* by interacting with *SmAREB1*. *Front. Plant Sci.* 8:1384. 10.3389/fpls.2017.01384 28848585 PMC5552723

[B21] Jiao L. Zhang Y. Lu J. (2017). Overexpression of a stress-responsive U-box protein gene *VaPUB* affects the accumulation of resistance related proteins in *Vitis vinifera* ‘Thompson Seedless’. *Plant Physiol. Biochem.* 112 53–63. 10.1016/j.plaphy.2016.12.019 28039816

[B22] Kong L. Cheng J. Zhu Y. Ding Y. Meng J. Chen Z. (2015). Degradation of the ABA co-receptor ABI1 by PUB12/13 U-box E3 ligases. *Nat. Commun.* 6 1–13. 10.1038/ncomms9630 26482222 PMC4667695

[B23] Kraft E. Stone S. L. Ma L. Su N. Gao Y. Lau O. S. (2005). Genome analysis and functional characterization of the E2 and RING-type E3 ligase ubiquitination enzymes of *Arabidopsis*. *Plant Physiol*. 139 1597–1611. 10.1104/pp.105.067983 16339806 PMC1310545

[B24] Letunic I. Bork P. (2021). Interactive Tree Of Life (iTOL) v5: an online tool for phylogenetic tree display and annotation. *Nucleic Acids Res.* 49 W293–W296. 10.1093/nar/gkab301 33885785 PMC8265157

[B25] Li K. Xing C. Yao Z. Huang X. (2017). *PbrMYB21*, a novel MYB protein of *Pyrus betulaefolia*, functions in drought tolerance and modulates polyamine levels by regulating arginine decarboxylase gene. *Plant Biotechnol. J.* 15 1186–1203. 10.1111/pbi.12708 28190292 PMC5552480

[B26] Liu Y. C. Wu Y. R. Huang X. H. Sun J. Xie Q. (2011). AtPUB19, a U-box E3 ubiquitin ligase, negatively regulates abscisic acid and drought responses in *Arabidopsis thaliana*. *Mol. Plant* 4 938–946. 10.1093/mp/ssr030 21502661 PMC3221247

[B27] Ma X. Mo B. Cao X. (2015). New players in ABA signaling: identification of PUB12/13 involved in degradation of ABA co-receptor ABI1. *Sci. China Life Sci.* 58:1173. 10.1007/s11427-015-4947-8 26511514

[B28] Melcher K. Ng L. M. Zhou X. E. Soon F. F. Xu Y. Suino-Powell K. M. (2009). A gate-latch-lock mechanism for hormone signalling by abscisic acid receptors. *Nature* 462 602–608. 10.1038/nature08613 19898420 PMC2810868

[B29] Mi H. Lazareva-Ulitsky B. Loo R. Kejariwal A. Vandergriff J. Rabkin S. (2005). The PANTHER database of protein families, subfamilies, functions and pathways. *Nucleic Acids Res*. 33 D284–D288. 10.1093/nar/gki078 15608197 PMC540032

[B30] Nystrom S. L. McKay D. J. (2021). Memes: a motif analysis environment in R using tools from the MEME Suite. *PLoS Computat. Biol.* 17:e1008991. 10.1371/journal.pcbi.1008991 34570758 PMC8496816

[B31] Osakabe Y. Yamaguchi-Shinozaki K. Shinozaki K. Tran L. S. P. (2014). ABA control of plant macroelement membrane transport systems in response to water deficit and high salinity. *New Phytol.* 202 35–49. 10.1111/nph.12613 24283512

[B32] Park S. Y. Fung P. Nishimura N. Jensen D. R. Fujii H. Zhao Y. (2009). Abscisic acid inhibits type 2C protein phosphatases via the PYR/PYL family of START proteins. *Science* 324 1068–1071. 10.1126/science.1173041 19407142 PMC2827199

[B33] Raab S. Drechsel G. Zarepour M. Hartung W. Koshiba T. Bittner F. (2009). Identification of a novel E3 ubiquitin ligase that is required for suppression of premature senescence in *Arabidopsis*. *Plant J.* 59 39–51. 10.1111/j.1365-313X.2009.03846.x 19309463

[B34] Richburg J. H. Myers J. L. Bratton S. B. (2014). The role of E3 ligases in the ubiquitin-dependent regulation of spermatogenesis. *Semin. Cell Dev. Biol*. 30 27–35. 10.1016/j.semcdb.2014.03.001 24632385 PMC4043869

[B35] Rombauts S. Déhais P. Van Montagu M. Rouzé P. (1999). PlantCARE, a plant *cis*-acting regulatory element database. *Nucleic Acids Res.* 27 295–296. 10.1093/nar/27.1.295 9847207 PMC148162

[B36] Rotin D. Kumar S. (2009). Physiological functions of the HECT family of ubiquitin ligases. *Nat. Rev. Mol. Cell Biol*. 10 398–409. 10.1038/nrm2690 19436320

[B37] Samuel M. A. Mudgil Y. Salt J. N. Delmas F. Ramachandran S. Chilelli A. (2008). Interactions between the S-domain receptor kinases and AtPUB-ARM E3 ubiquitin ligases suggest a conserved signaling pathway in *Arabidopsis*. *Plant Physiol*. 147 2084–2095. 10.1104/pp.108.123380 18552232 PMC2492606

[B38] Santner A. Estelle M. (2010). The ubiquitin-proteasome system regulates plant hormone signaling. *Plant J.* 61 1029–1040. 10.1111/j.1365-313X.2010.04112.x 20409276 PMC3066055

[B39] Schaart J. G. Dubos C. Romero De La Fuente I. van Houwelingen A. M. de Vos R. C. Jonker H. H. (2013). Identification and characterization of MYB-bHLH-WD40 regulatory complexes controlling proanthocyanidin biosynthesis in strawberry (*Fragaria* × *ananassa*) fruits. *New Phytol.* 197 454–467. 10.1111/nph.12017 23157553

[B40] Seo D. H. Ryu M. Y. Jammes F. Hwang J. H. Turek M. Kang B. G. (2012). Roles of four *Arabidopsis* U-box E3 ubiquitin ligases in negative regulation of abscisic acid-mediated drought stress responses. *Plant Physiol*. 160 556–568. 10.1104/pp.112.202143 22829319 PMC3440228

[B41] Serrano I. Campos L. Rivas S. (2018). Roles of E3 ubiquitin-ligases in nuclear protein homeostasis during plant stress responses. *Front. Plant Sci.* 9:139. 10.3389/fpls.2018.00139 29472944 PMC5809434

[B42] Shannon P. Markiel A. Ozier O. Baliga N. S. Wang J. T. Ramage D. (2003). Cytoscape: a software environment for integrated models of biomolecular interaction networks. *Genome Res.* 13 2498–2504. 10.1101/gr.1239303 14597658 PMC403769

[B43] Sharma B. Taganna J. (2020). Genome-wide analysis of the U-box E3 ubiquitin ligase enzyme gene family in tomato. *Sci. Rep*. 10:9581. 10.1038/s41598-020-66553-1 32533036 PMC7293263

[B44] Shen Y. Sun T. Pan Q. Anupol N. Chen H. Shi J. (2019). RrMYB5- and RrMYB10- regulated flavonoid biosynthesis plays a pivotal role in feedback loop responding to wounding and oxidation in *Rosa rugosa*. *Plant Biotechnol. J.* 17 2078–2095. 10.1111/pbi.13123 30951245 PMC6790370

[B45] Shi M. Hua Q. Kai G. (2021). Comprehensive transcriptomic analysis in response to abscisic acid in *Salvia miltiorrhiza*. *Plant Cell Tissue Organ. Culture (PCTOC)* 147 389–404. 10.1007/s11240-021-02135-x

[B46] Shi M. Luo X. Ju G. Li L. Huang S. Zhang T. (2016). Enhanced diterpene tanshinone accumulation and bioactivity of transgenic *Salvia miltiorrhiza* hairy roots by pathway engineering. *J. Agric. Food Chem*. 64 2523–2530. 10.1021/acs.jafc.5b04697 26753746

[B47] Shu K. Yang W. (2017). E3 ubiquitin ligases: ubiquitous actors in plant development and abiotic stress responses. *Plant Cell Physiol*. 58 1461–1476. 10.1093/pcp/pcx071 28541504 PMC5914405

[B48] Song J. Mo X. Yang H. Yue L. Song J. Mo B. (2017). The U-box family genes in *Medicago truncatula*: key elements in response to salt, cold, and drought stresses. *PLoS One* 12:e0182402. 10.1371/journal.pone.0182402 28771553 PMC5542650

[B49] Stone S. L. Hauksdóttir H. Troy A. Herschleb J. Kraft E. Callis J. (2005). Functional analysis of the RING-Type ubiquitin ligase family of *Arabidopsis*. *Plant Physiol*. 137 13–30. 10.1104/pp.104.052423 15644464 PMC548835

[B50] Tamura K. Stecher G. Peterson D. Filipski A. Kumar S. (2013). MEGA6: molecular evolutionary genetics analysis version 6.0. *Mol. Biol. Evol.* 30 2725–2729. 10.1093/molbev/mst197 24132122 PMC3840312

[B51] Tian M. Xie Q. (2013). Non-26S proteasome proteolytic role of ubiquitin in plant endocytosis and endosomal trafficking. *J. Integrat. Plant Biol*. 55 54–63. 10.1111/jipb.12007 23137267

[B52] Trujillo M. (2018). News from the PUB: plant U-box type E3 ubiquitin ligases. *J. Exp. Bot*. 69 371–384. 10.1093/jxb/erx411 29237060

[B53] Trujillo M. Shirasu K. (2010). Ubiquitination in plant immunity. *Curr. Opin. Plant Biol.* 13 402–408. 10.1016/j.pbi.2010.04.002 20471305

[B54] Umezawa T. Nakashima K. Miyakawa T. Kuromori T. Tanokura M. Shinozaki K. (2010). Molecular basis of the core regulatory network in ABA responses: sensing, signaling and transport. *Plant Cell Physiol.* 51 1821–1839. 10.1093/pcp/pcq156 20980270 PMC2978318

[B55] Vandereyken K. Van Leene J. De Coninck B. Cammue B. (2018). Hub protein controversy: taking a closer look at plant stress response hubs. *Front. Plant Sci*. 9:694. 10.3389/fpls.2018.00694 29922309 PMC5996676

[B56] Wang C. Song B. Dai Y. Zhang S. Huang X. (2021). Genome-wide identification and functional analysis of U-box E3 ubiquitin ligases gene family related to drought stress response in Chinese white pear (*Pyrus bretschneideri*). *BMC Plant Biol*. 21:235. 10.1186/s12870-021-03024-3 34039263 PMC8152096

[B57] Wang J. Liu S. Liu H. Chen K. Zhang P. (2019). PnSAG1, an E3 ubiquitin ligase of the Antarctic moss *Pohlia nutans*, enhanced sensitivity to salt stress and ABA. *Plant Physiol. Biochem*. 141 343–352. 10.1016/j.plaphy.2019.06.002 31207495

[B58] Wang K. Yang Q. Lanhuang B. Lin H. Shi Y. Dhanasekaran S. (2020). Genome-wide investigation and analysis of U-box Ubiquitin-Protein ligase gene family in apple: expression profiles during *Penicillium expansum* infection process. *Physiol. Mol. Plant Pathol*. 111:101487. 10.1016/j.pmpp.2020.101487

[B59] Wang N. Liu Y. Cong Y. Wang T. Zhong X. Yang S. (2016). Genome-wide identification of soybean U-box E3 ubiquitin ligases and roles of GmPUB8 in negative regulation of drought stress response in Arabidopsis. *Plant Cell Physiol*. 57 1189–1209. 10.1093/pcp/pcw068 27057003

[B60] Wang Z. Tian X. Zhao Q. Liu Z. Li X. Ren Y. (2018). The E3 ligase DROUGHT HYPERSENSITIVE negatively regulates cuticular wax biosynthesis by promoting the degradation of transcription factor ROC4 in rice. *Plant Cell* 30 228–244. 10.1105/tpc.17.00823 29237723 PMC5810576

[B61] Wu Y. Zhang Y. Li L. Guo X. Wang B. Cao X. (2018). AtPAP1 interacts with and activates SmbHLH51, a positive regulator to phenolic acids biosynthesis in *Salvia miltiorrhiza*. *Front. Plant Sci.* 9:1687. 10.3389/fpls.2018.01687 30515184 PMC6255977

[B62] Xu G. Guo C. Shan H. Kong H. (2012). Divergence of duplicate genes in exon-intron structure. *Proc. Natl. Acad. Sci. U.S.A*. 109 1187–1192. 10.1073/pnas.1109047109 22232673 PMC3268293

[B63] Xu H. Song J. Luo H. Zhang Y. Li Q. Zhu Y. (2016). Analysis of the genome sequence of the medicinal plant *Salvia miltiorrhiza*. *Mol. Plant* 9:949. 10.1016/j.molp.2016.03.010 27018390 PMC5517341

[B64] Xu W. Dubos C. Lepiniec L. (2015). Transcriptional control of flavonoid biosynthesis by MYB-bHLH-WDR complexes. *Trends Plant Sci.* 20 176–185. 10.1016/j.tplants.2014.12.001 25577424

[B65] Yee D. Goring D. R. (2009). The diversity of plant U-box E3 ubiquitin ligases: from upstream activators to downstream target substrates. *J. Exp. Bot.* 60 1109–1121. 10.1093/jxb/ern369 19196749

[B66] Yu H. Jiang M. Xing B. Liang L. Zhang B. Liang Z. (2020). Systematic analysis of Kelch Repeat F-box (KFB) protein family and identification of phenolic acid regulation members in *Salvia miltiorrhiza* Bunge. *Genes* 11:557. 10.3390/genes11050557 32429385 PMC7288277

[B67] Yu Y. H. Li X. Z. Guo D. L. Zhang H. L. Li G. R. Li X. Q. (2016). Genome-wide identification and analysis of the U-box family of E3 ligases in grapevine. *Russian J. Plant Physiol.* 63 835–848. 10.1134/S1021443716050186

[B68] Zeng L. R. Park C. H. Venu R. C. Gough J. Wang G. L. (2008). Classification, expression pattern, and E3 ligase activity assay of rice U-box-containing proteins. *Mol. Plant* 1 800–815. 10.1093/mp/ssn044 19825583

[B69] Zhai R. Wang Z. Zhang S. Meng G. Song L. Wang Z. (2016). Two MYB transcription factors regulate flavonoid biosynthesis in pear fruit (*Pyrus bretschneideri* Rehd.). *J. Exp. Bot.* 67 1275–1284. 10.1093/jxb/erv524 26687179

[B70] Zhang X. Xu Z. Yu X. Zhao L. Zhao M. Han X. (2019). Identification of two novel R2R3-MYB transcription factors, *PsMYB114L* and *PsMYB12L*, related to anthocyanin biosynthesis in *Paeonia suffruticosa*. *Int. J. Mol. Sci.* 20:1055. 10.3390/ijms20051055 30823465 PMC6429501

[B71] Zhou L. Zuo Z. Chow M. S. S. (2005). Danshen: an overview of its chemistry, pharmacology, pharmacokinetics, and clinical use. *J. Clin. Pharmacol*. 45 1345–1359. 10.1177/0091270005282630 16291709

[B72] Zhou W. Huang F. Li S. Wang Y. Zhou C. Shi M. (2016). Molecular cloning and characterization of two 1-deoxy-d-xylulose-5-phosphate synthase genes involved in tanshinone biosynthesis in *Salvia miltiorrhiza*. *Mol. Breed*. 36 1–12. 10.1007/s11032-016-0550-3

[B73] Zhou W. Huang Q. Wu X. Zhou Z. Ding M. Shi M. (2017). Comprehensive transcriptome profiling of *Salvia miltiorrhiza* for discovery of genes associated with the biosynthesis of tanshinones and phenolic acids. *Sci. Rep.* 7:10554. 10.1038/s41598-017-10215-2 28874707 PMC5585387

[B74] Zhou W. Shi M. Deng C. Lu S. Huang F. Wang Y. (2021). The methyl jasmonate-responsive transcription factor SmMYB1 promotes phenolic acid biosynthesis in *Salvia miltiorrhiza*. *Hortic. Res*. 8 1–13. 10.1038/s41438-020-00443-5 33384411 PMC7775463

