# Supplementary figures and images for: Genome-Wide Analysis of U-box E3 Ubiquitin Ligase Family in Response to ABA Treatment in Salvia miltiorrhiza
Source: Front Plant Sci. 2022 Feb 9;13:829447. doi: 10.3389/fpls.2022.829447 (PMC8863962; doi:10.3389/fpls.2022.829447)

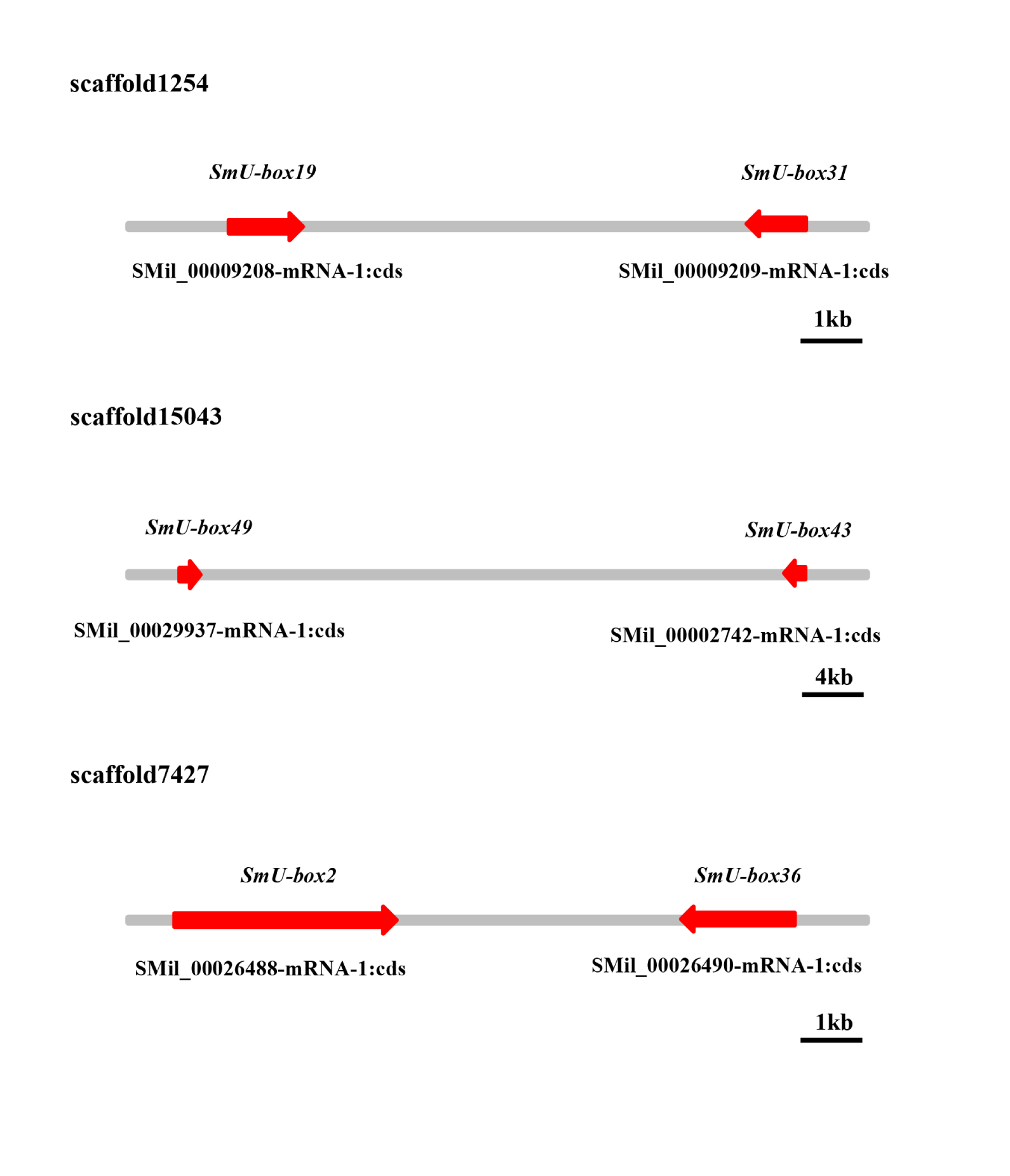

Supplement: Supplementary Figure 1 — The location of UBE3 genes in S. miltiorrhiza genome. [file Image_1.TIF]

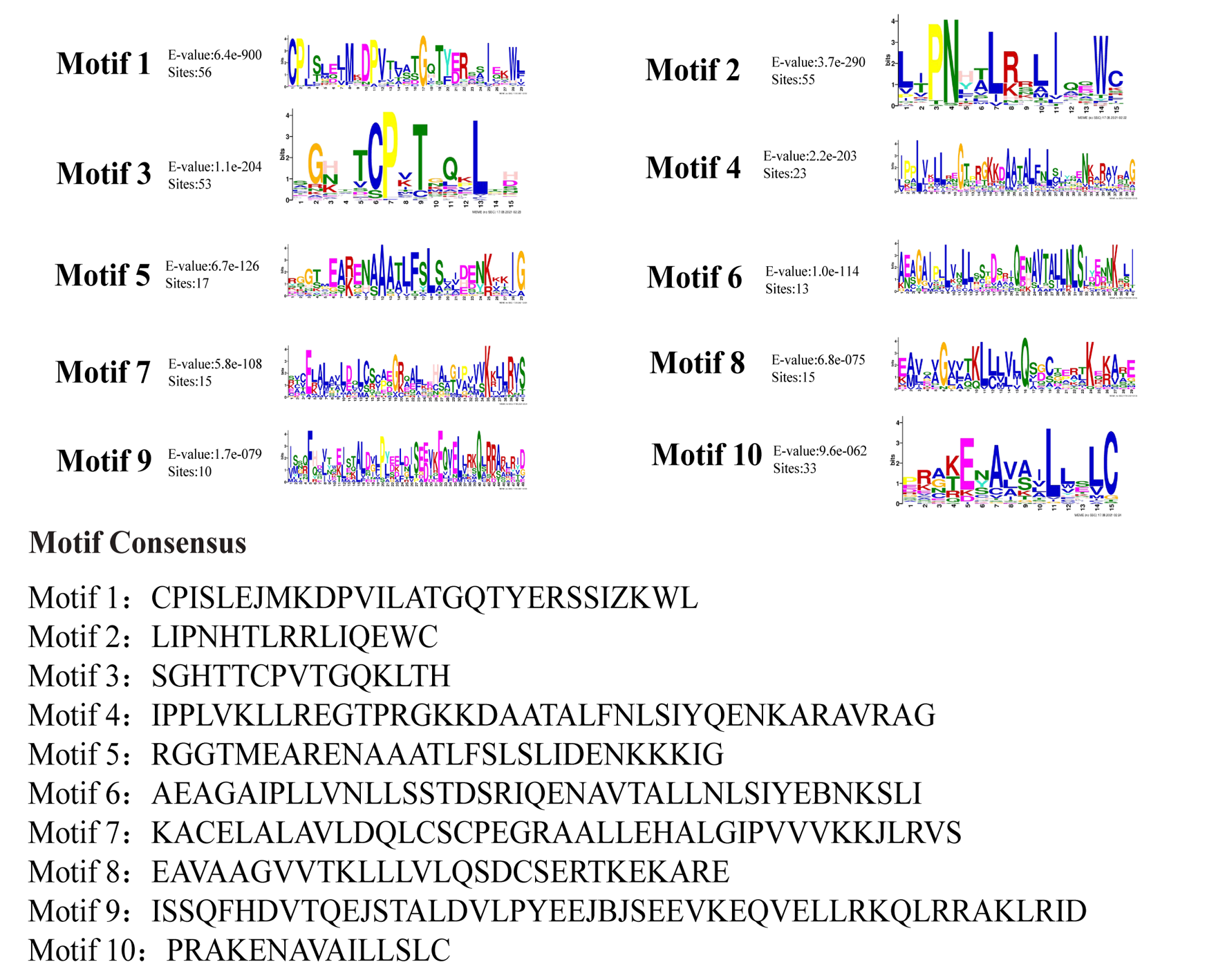

Supplement: Supplementary Figure 2 — Ten motifs identified using MEME suit for U-box gene family. [file Image_2.TIF]

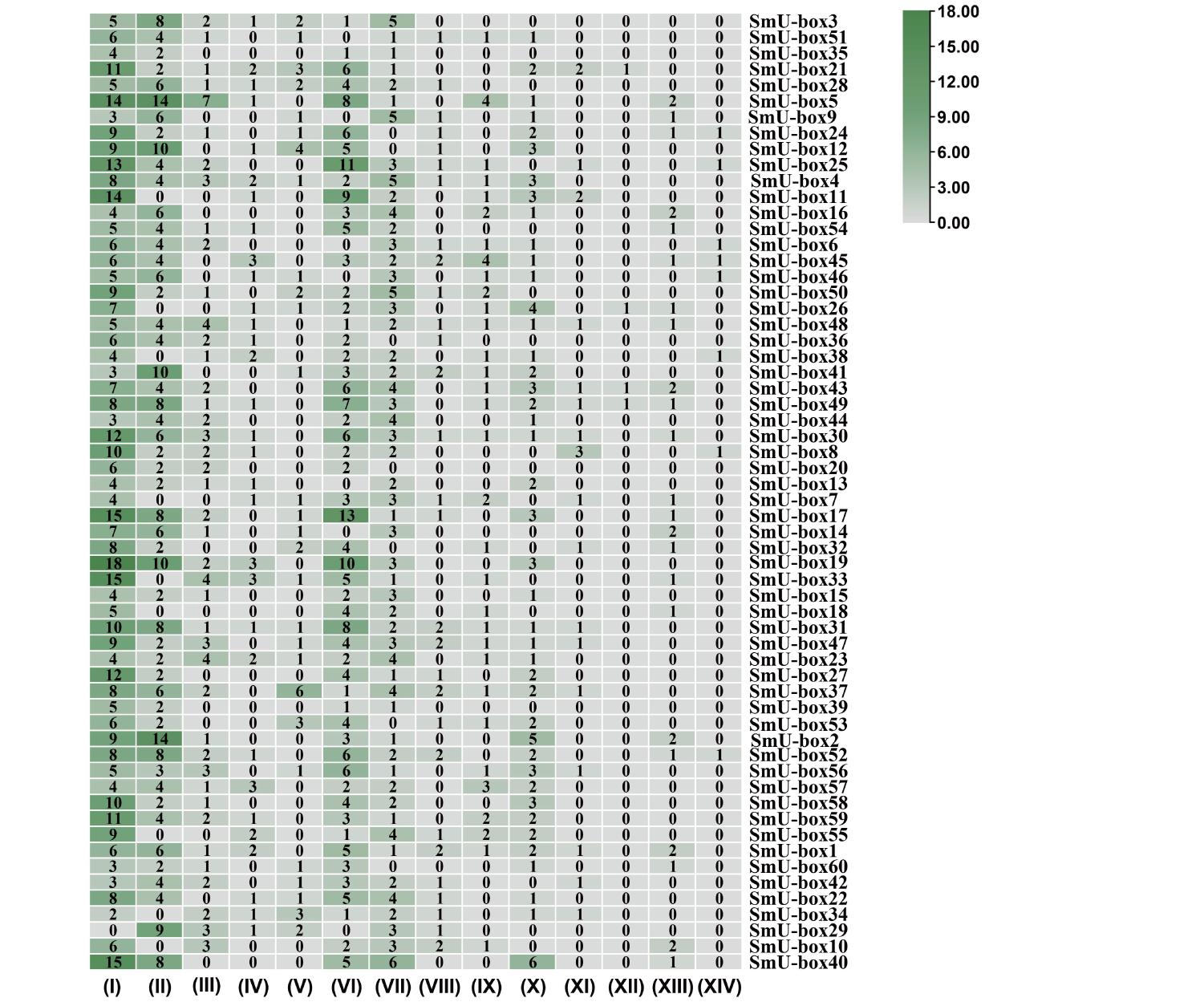

Supplement: Supplementary Figure 3 — The number of 14 cis-acting elements in each putative promoter of UBE3 genes. The color scale at the bottom indicated the number of cis-acting elements. [file Image_3.TIF]

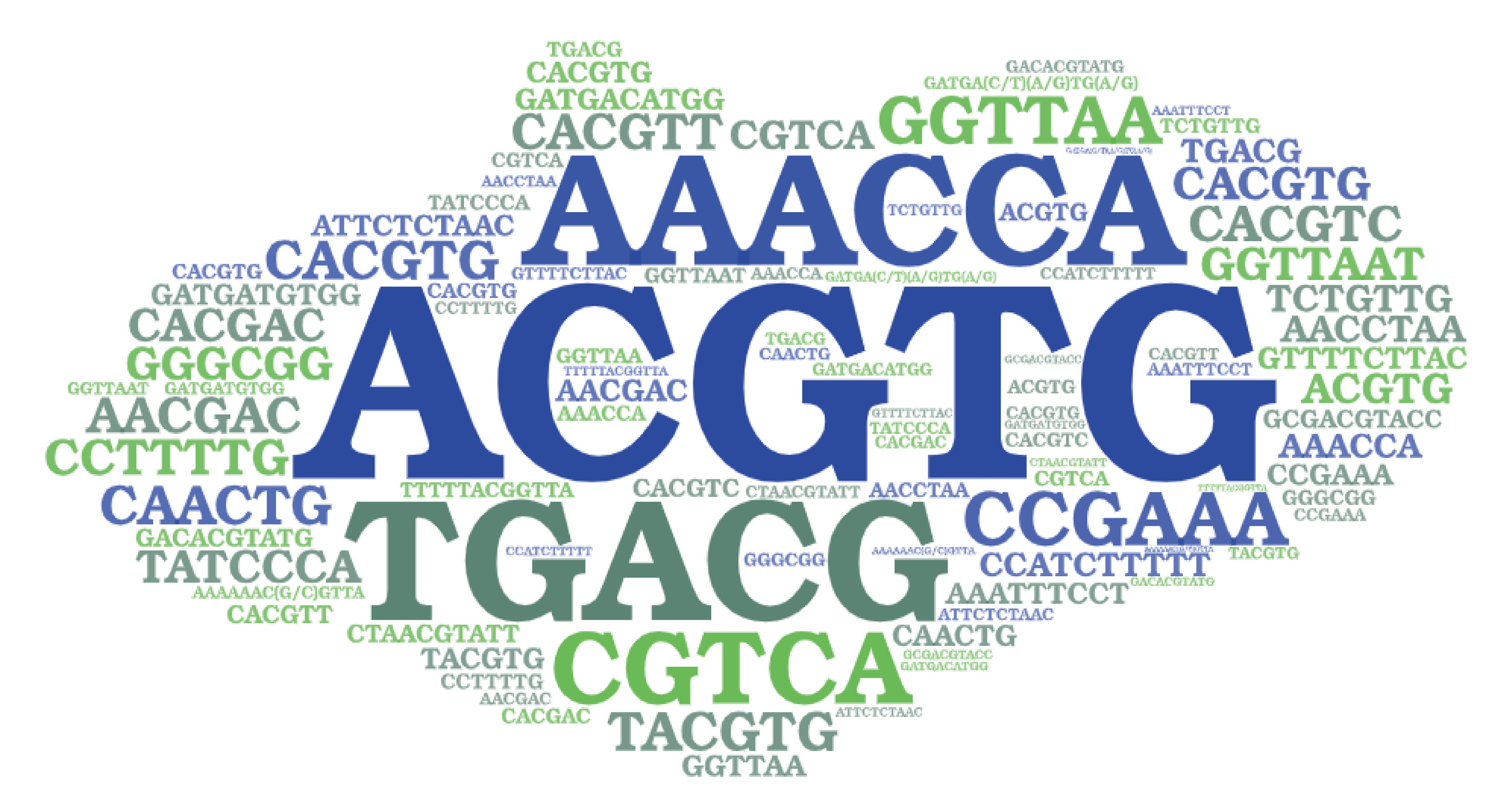

Supplement: Supplementary Figure 4 — The word cloud image of cis-acting elements in the promoter of 60 UBE3 genes. [file Image_4.TIF]

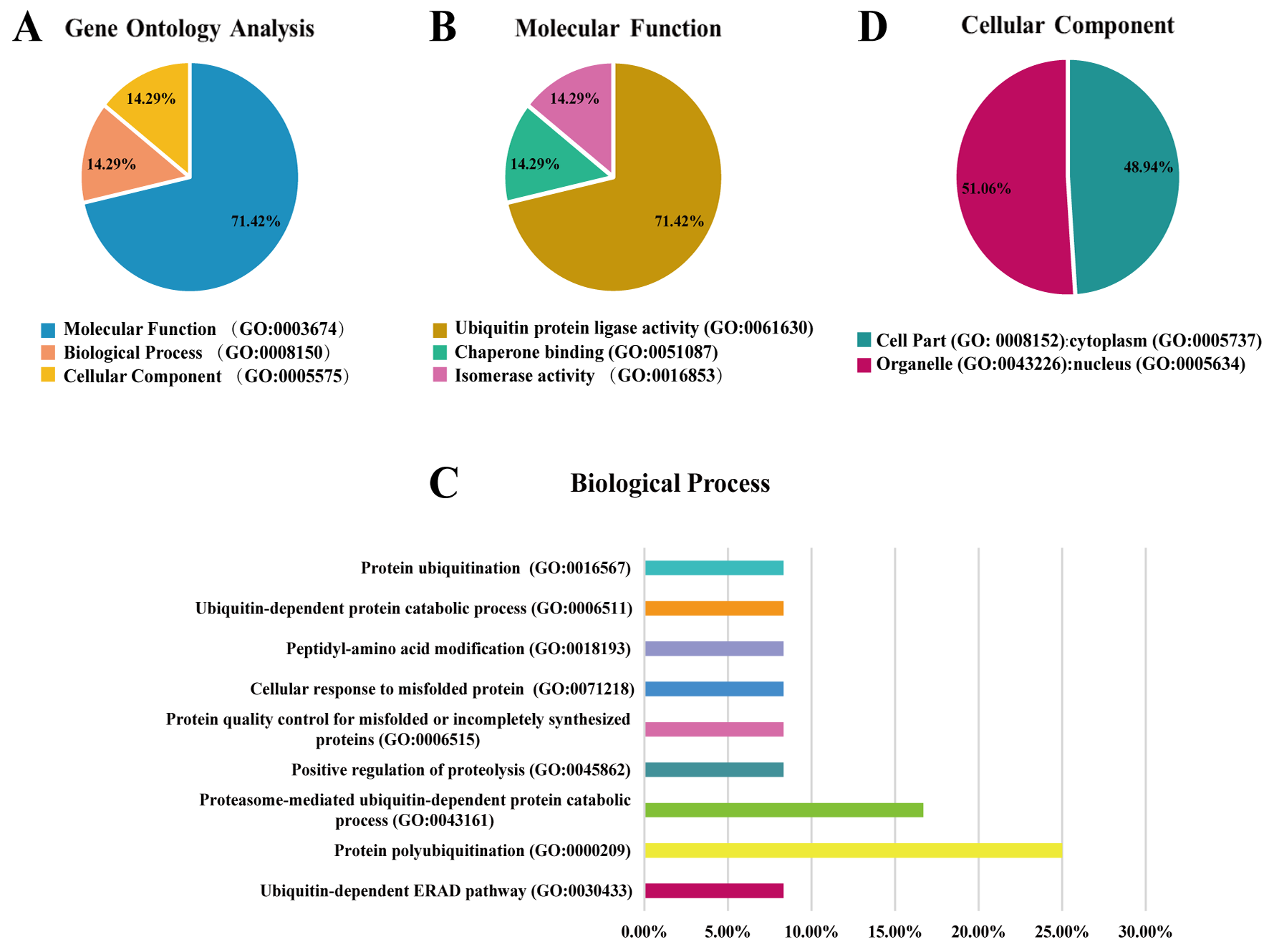

Supplement: Supplementary Figure 5 — Gene ontology analysis of 60 UBE3 genes in S. miltiorrhiza. (A) Gene ontology analysis of the identified UBE3 genes in S. miltiorrhiza. (B) The molecular function of UBE3 genes in S. miltiorrhiza. (C) The biological process of UBE3 genes in S. miltiorrhiza. (D) The cellular component of UBE3 genes in S. miltiorrhiza. [file Image_5.TIF]

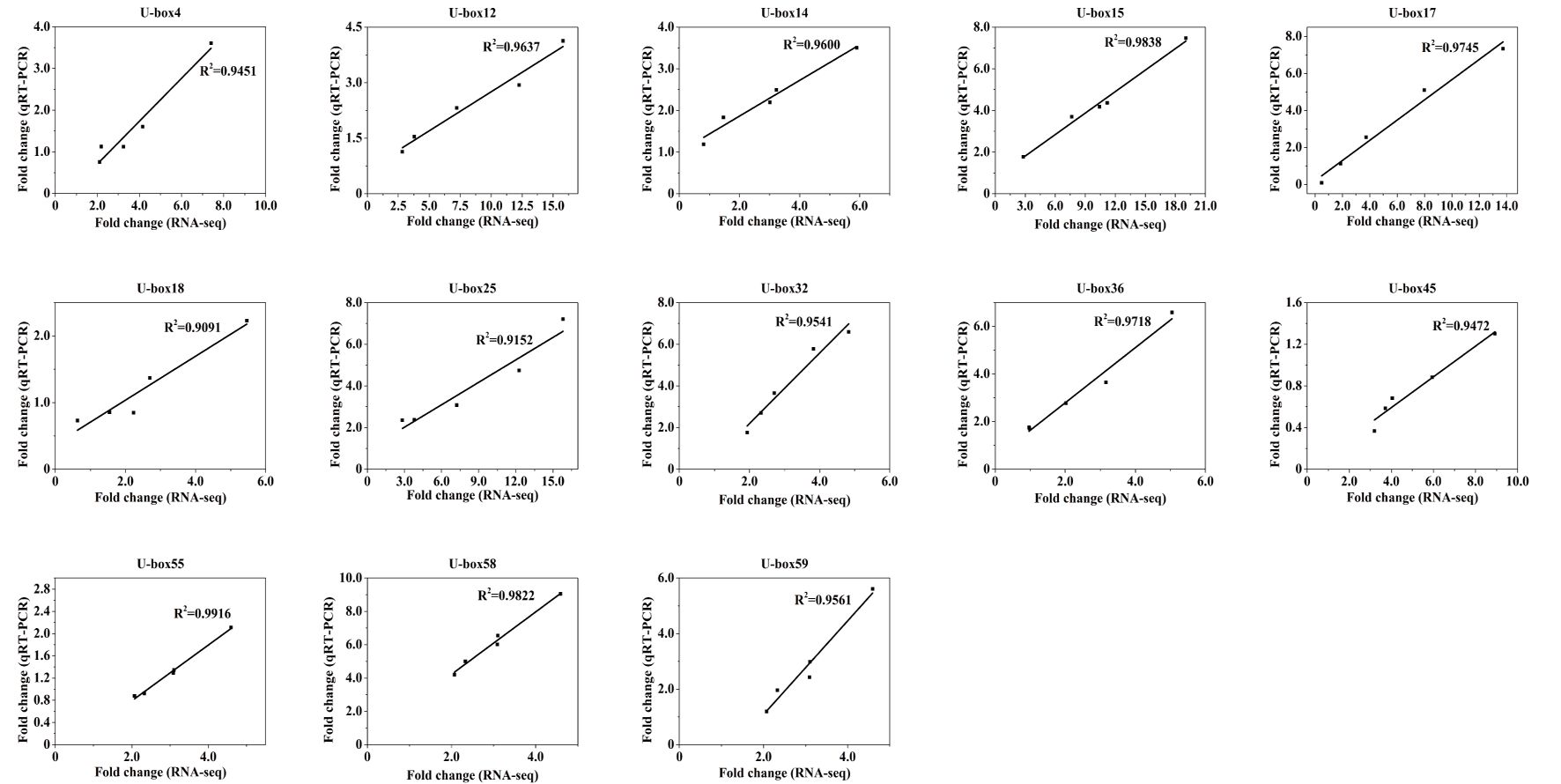

Supplement: Supplementary Figure 6 — Correlation of gene expression results obtained from qRT-PCR and RNA-seq. [file Image_6.TIF]

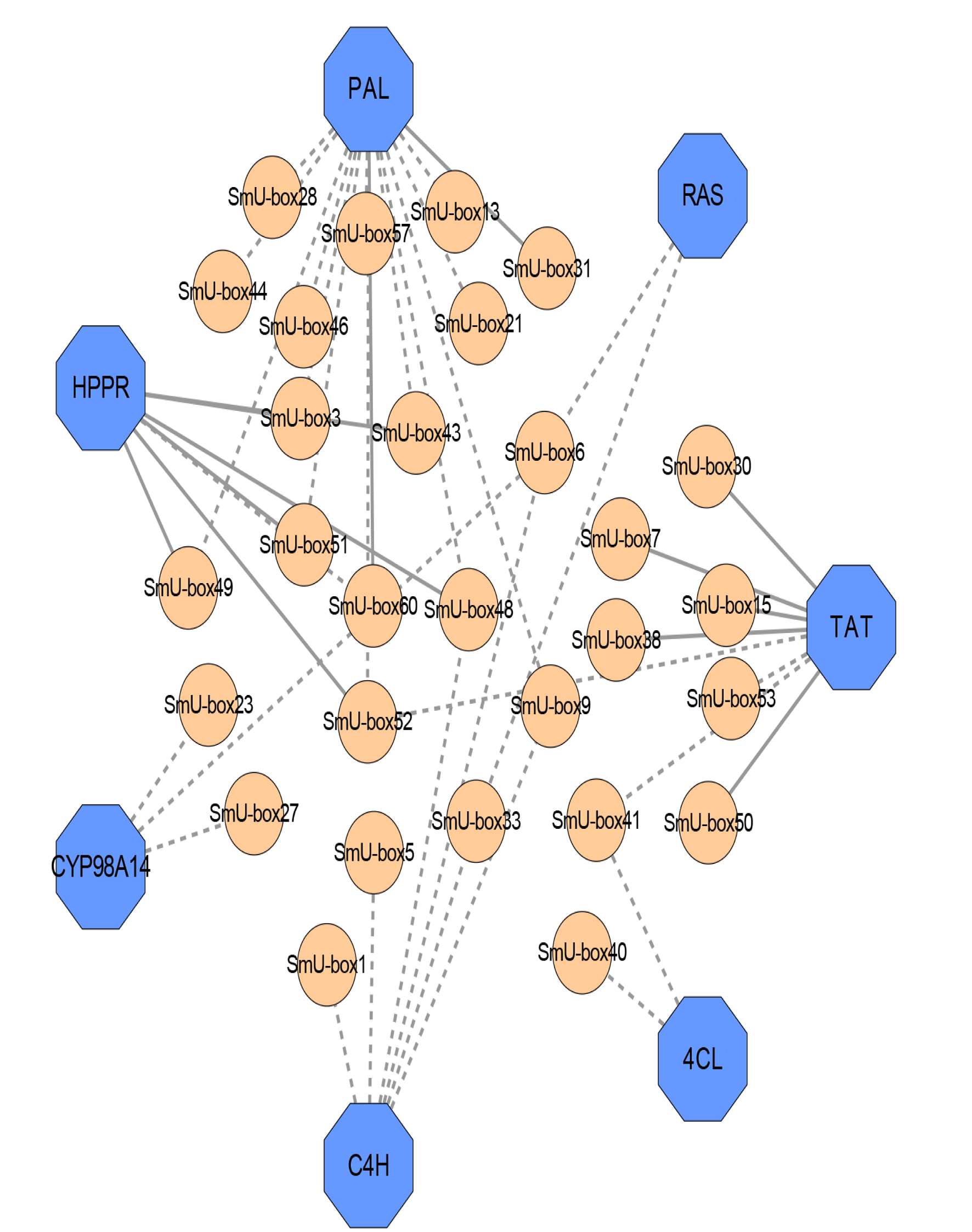

Supplement: Supplementary Figure 7 — The co-expression network of UBE3 genes and phenolic acids biosynthetic genes. [file Image_7.TIF]

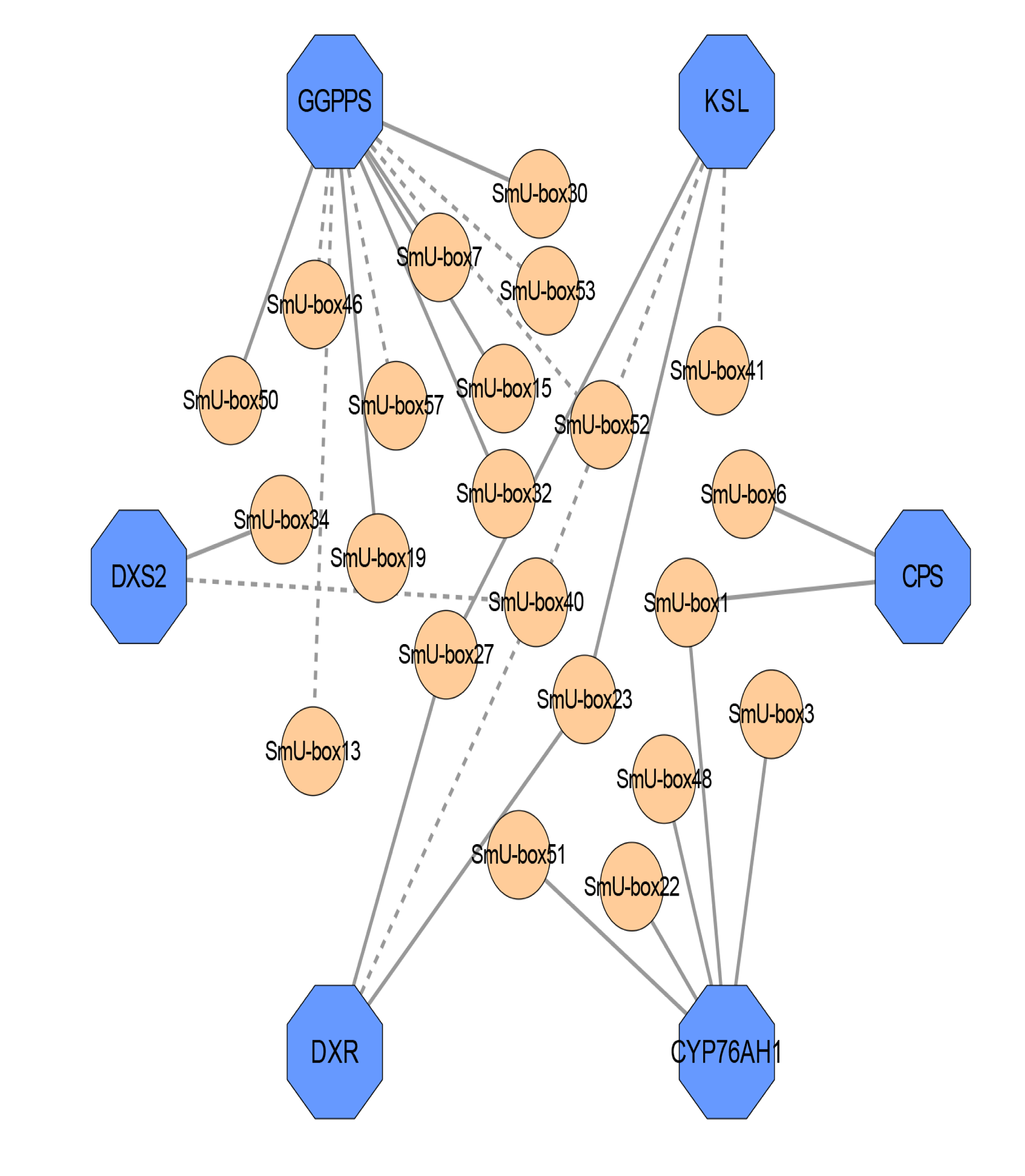

Supplement: Supplementary Figure 8 — The co-expression network of UBE3 genes and tanshinone biosynthetic genes. [file Image_8.TIF]
