# Supplementary material for: Genome-Wide Analysis of U-box E3 Ubiquitin Ligase Family in Response to ABA Treatment in Salvia miltiorrhiza
Source: Front Plant Sci. 2022 Feb 9;13:829447. doi: 10.3389/fpls.2022.829447 (PMC8863962; doi:10.3389/fpls.2022.829447)
Supplement: Supplementary file 9 [file Table_1.DOCX]

**Table S1. Primer sequences used in qRT-PCR analysis.**

| **Primers** | **Sequences (5’-3’)** |
| --- | --- |
| SmU-box4-F | ATCGAGAGCTCCGTACAAGG |
| SmU-box4-R | TGCCCATCTCCTTCACCAAT |
| SmU-box12-F | GTTCATGATCAGGCGGGTTC |
| SmU-box12-R | ACTTCCACGTCGTAGTCCTC |
| SmU-box14-F | ATGAGGAGACGAGCCGCAACC |
| SmU-box14-R | AATCCCATCTCCGCCAAACG |
| SmU-box15-F | CCTCCGACAACCTCACCAACA |
| SmU-box15-R | ATGTCTTCCGCCTTCAAAAT |
| SmU-box17-F | CTCTGCAGACAAGGGTGAGA |
| SmU-box17-R | CATGCAGAGCTTCCAAAGGG |
| SmU-box18-F | AAGATCCTGAGGGTTTCGCA |
| SmU-box18-R | TCTCTCACTAGCCTCCACCT |
| SmU-box25-F | TGGTGGTAGGGACGAATGAG |
| SmU-box25-R | TTACGCTGCAATGCCTGATC |
| SmU-box32-F | AGGTGCTGGTGAGGATAATGGA |
| SmU-box32-R | TCCAGCAGAGATTTGAAGAAGC |
| SmU-box36-F | GAACCTCTCCATCCACGACA |
| SmU-box36-R | ATAACGTGAAGAGGGTCGCA |
| SmU-box45-F | CCTCTGCCCGTTCCCTTTTT |
| SmU-box45-R | CTCCGAGAGGAAAGAAACAG |
| SmU-box55-F | GACCTCTTCCACTCACCCAA |
| SmU-box55-R | TCTCAACAACAGGGCAATGC |
| SmU-box58-F | GATACAGTCGGTTGCGGGTTG |
| SmU-box58-R | GCACTTGCCTCCTGTTTCGT |
| SmU-box59-F | GATACAGTCGGTTGCGGGTTG |
| SmU-box59-R | TCCCAAACAGTTACTATGCC |
| Actin F | AGCACCGAGCAGCATGAAGATT |
| Actin R | AGCAAAGCAGCGAACGAAGAGT |
