# Supplementary material for: Genome-Wide Analysis of U-box E3 Ubiquitin Ligase Family in Response to ABA Treatment in Salvia miltiorrhiza
Source: Front Plant Sci. 2022 Feb 9;13:829447. doi: 10.3389/fpls.2022.829447 (PMC8863962; doi:10.3389/fpls.2022.829447)
Supplement: Supplementary file 11 [file Table_3.DOC]

**Table S3. Domain organizations of UBE3 proteins.**

| **Type** | **Domain organization**  **(N-terminus to C-terminus)** | **Number** |
| --- | --- | --- |
| I | U-box only | 3 |
| II | Kinase+U-box | 7 |
| III | U-box+ARM | 21 |
| IV | U-box+GKL-box | 21 |
| V | U-box+WD40 | 7 |
| VI | UFD2+U-box | 1 |
